# Fluoxetine promotes IL-10–dependent metabolic defenses to protect from sepsis-induced lethality

**DOI:** 10.1126/sciadv.adu4034

**Published:** 2025-02-14

**Authors:** Robert M. Gallant, Karina K. Sanchez, Emeline Joulia, Jessica M. Snyder, Christian M. Metallo, Janelle S. Ayres

**Affiliations:** ^1^ Molecular and Systems Physiology Laboratory, Salk Institute for Biological Studies, 10010 N. Torrey Pines Road, La Jolla, CA 92037, USA.; ^2^ Division of Biological Sciences, University of California, San Diego, La Jolla, CA 92037, USA.; ^3^ NOMIS Center for Immunobiology and Microbial Pathogenesis, Salk Institute for Biological Studies, La Jolla, CA 92037, USA.; ^4^ Gene Expression Laboratory, Salk Institute for Biological Studies, 10010 N. Torrey Pines Road, La Jolla, CA 92037, USA.; ^5^ Howard Hughes Medical Institute, Salk Institute for Biological Studies, La Jolla, CA 92037, USA.; ^6^ Molecular and Cell Biology Laboratory, Salk Institute for Biological Studies, La Jolla, CA 92037, USA.; ^7^ Department of Comparative Medicine, School of Medicine, University of Washington, Seattle, WA 98195, USA.; ^8^ Department of Bioengineering, University of California San Diego, La Jolla, CA 92037, USA.

## Abstract

Selective serotonin reuptake inhibitors (SSRIs) are some of the most prescribed drugs in the world. While they are used for their ability to increase serotonergic signaling in the brain, SSRIs are also known to have a broad range of effects beyond the brain, including immune and metabolic effects. Recent studies have demonstrated that SSRIs are protective in animal models and humans against several infections, including sepsis and COVID-19; however, the mechanisms underlying this protection are largely unknown. Here, we mechanistically link two previously described effects of the SSRI fluoxetine in mediating protection against sepsis. We show that fluoxetine-mediated protection is independent of peripheral serotonin and instead increases levels of circulating interleukin-10 (IL-10). IL-10 is necessary for protection from sepsis-induced hypertriglyceridemia, preventing cardiac effects including impairment of glucose oxidation, ectopic lipid accumulation, ventricular stretch and possibly cardiac failure. Our work reveals a beneficial “off-target” effect of fluoxetine, and reveals a protective immunometabolic defense mechanism with therapeutic potential.

## Teaser

An SSRI protects from infection induced lethality by promoting immune-dependent metabolic defenses.

## INTRODUCTION

Host defense strategies against pathogens can be broadly categorized based on their effects on pathogen fitness (*1*). Antagonistic defenses protect the host by having a negative impact on pathogen fitness. They include both resistance defense mechanisms that kill pathogens and avoidance mechanisms that prevent exposure to pathogens. In contrast, cooperative defenses facilitate host adaptation to the infected state and protect the host while having a neutral to positive effect on pathogen fitness. They include both antivirulence mechanisms that block host and microbial-derived pathogenic signals and disease tolerance mechanisms that limit host susceptibility to physiological dysfunction and damage (*2*). Treatment strategies for infectious diseases will follow these same principles. Drug interventions can have antimicrobial actions via direct effects on the pathogen, such as antibiotics, or indirectly by heightening the host immune response to the infection. They can also promote host-pathogen cooperation by acting to neutralize/block pathogenic signals that can cause damage, for example, as seen with the anti-inflammatory actions of steroids, or promote disease tolerance by limiting susceptibility to physiological dysfunction/damage, such as the use of fluid replacement or vasoconstrictors to raise blood pressure in critical illness. Most of the infectious disease treatments work to rid us of pathogens. However, for some infectious diseases, including sepsis, the host response to the infection is more damaging than the pathogen, and thus, antimicrobial-based therapeutics are often insufficient for patient survival within these contexts (*3*). Host-targeted therapeutic approaches that neutralize or detoxify host-derived pathogenic signals and that protect from physiological dysfunction and damage in response to pathogenic signals are needed.

Sepsis is defined as a life-threatening organ dysfunction caused by a dysregulated host response to infection (*4*). The most obvious way to target the host response in a septic patient is to suppress the inflammatory response. Numerous clinical trials have been performed with neutralizing antibodies that target pro-inflammatory cytokines; however, these approaches have been met with little success (*5*–*9*). One explanation for why this strategy has been largely unsuccessful is that these strategies can immunocompromise the patient, making their primary infections more difficult to control and/or rendering them more susceptible to secondary infections. A second possible explanation is that timing is critical, and these interventions must be administered before damage occurs (*10*). In addition, sepsis is a multifactorial syndrome with many derangements in host physiology beyond immune overactivation, including dysfunction of the metabolic response of the patient. Host-directed therapeutics that can control the degree and duration of an immune response to allow pathogen killing but prevent the escalation of the response to a cytokine storm and that also promote metabolic adaptation to the infected state may offer more therapeutic benefit than strategies that focus only on blocking the pro-inflammatory response.

Selective serotonin reuptake inhibitors (SSRIs) are some of the most widely prescribed drugs with more than 13% of adults using these antidepressant medications between 2015 and 2018 (*11*). SSRIs were originally developed in the 1970s for their ability to prevent serotonin reuptake in the synaptic cleft of serotonergic neurons (*12*). It is now recognized that SSRIs also have a wide range of peripheral effects including regulation of immune and metabolic processes (*13*, *14*). Furthermore, SSRIs have been shown to protect against sepsis in animal models (*15*) and improve outcomes in patients infected with severe acute respiratory syndrome coronavirus 2 (*16*). The mechanisms underlying these protective effects are unclear. SSRIs have been reported to have anti-inflammatory effects, which suggest that they may protect against overwhelming inflammatory responses and cytokine storm (*17*, *18*). They have also been reported to regulate aspects of systemic metabolism that are dysregulated during sepsis and other inflammatory states including lipid metabolism (*19*–*21*). It remains unclear whether the anti-inflammatory effects of SSRIs are somehow related to the drug’s effect on lipid metabolism or if these are independent effects of SSRIs that may regulate host survival.

Here, we examined how the SSRI, fluoxetine, regulates survival and disease progression in a mouse model of sepsis. We found that fluoxetine pretreatment promotes both resistance and cooperative defenses in a peripheral serotonin-independent manner. Instead, fluoxetine increases circulating levels of interleukin-10 (IL-10), which in turn protects from sepsis induced hypertriglyceridemia, preventing cardiac effects including impairment of glucose oxidation, ectopic lipid accumulation, ventricular stretch, and possibly cardiac failure. Our study mechanistically links the anti-inflammatory and metabolic effects of an SSRI and demonstrates that fluoxetine can be used as a prophylactic to protect from sepsis induced lethality by orchestrating protective immunometabolic mechanisms, which may be leveraged and further explored by future studies.

## RESULTS

### Prophylactic fluoxetine protects from sepsis-induced disease and mortality

**Fig. 1.**
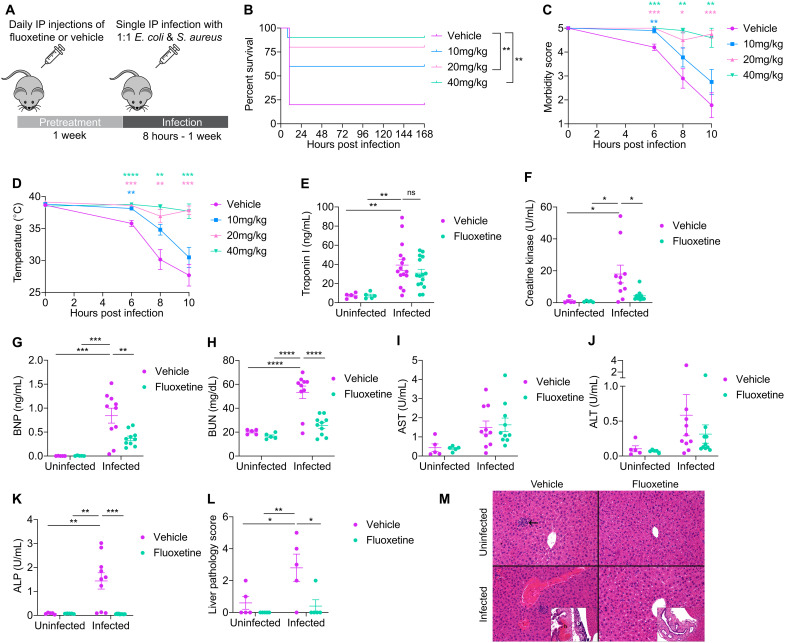
Fluoxetine pretreatment protects from sepsis-induced disease and mortality. (**A**) Schematic of experimental approach. C57BL/6J mice were treated for 1 week with daily intraperitoneal vehicle or fluoxetine injections then intraperitoneally infected with a 1:1 mixture of *E. coli* and *S. aureus*. (**B** to **D**) (B) Survival, (C) morbidity, and (D) temperature of vehicle- and fluoxetine-pretreated mice infected with polymicrobial sepsis. *n* = 10 per condition, one representative experiment shown. For survival, log-rank analysis. For morbidity and temperature, two-way analysis of variance (ANOVA) with Dunnett’s multiple comparisons test. (**E** to **K**) Levels of organ damage markers in serum of vehicle- and fluoxetine-pretreated mice infected with polymicrobial sepsis. Infected samples collected 8 to 10 hours post infection. *n* = 5 to 15 per condition, two to three independent experiments combined. (E) Troponin I. (F) Creatine kinase. (G) Brain natriuretic peptide (BNP). (H) Blood urea nitrogen (BUN). (I) Aspartate transaminase (AST). (J) Alanine transaminase (ALT). (K) Alkaline phosphatase (ALP). Two-way ANOVA with Tukey’s multiple comparisons test. (**L** to **M**) Liver pathology of vehicle- and fluoxetine-treated mice infected with polymicrobial sepsis. Infected samples collected 10 hours post infection. (L) Liver pathology score is combined necrosis, hemorrhage, and congestion scores from fig. S1 (B to D). (M) Representative images. Arrow shows a microgranuloma/microabscess, which were seen occasionally in mice from all groups and represent an incidental background lesion. Inset of the worst infected vehicle animal shows acute hemorrhage (h) and bacteria (b) in the region surrounding the gall bladder, compared to the worst fluoxetine-infected animal showing no hemorrhage or bacteria. *n* = 5 per condition, one independent experiment shown. There is mild microvesicular cytoplasmic vacuolation in the fluoxetine-treated mice. Two-way ANOVA with Tukey’s multiple comparisons test. In all panels, data represent means ± SEM. *P < 0.05, **P < 0.01, ***P < 0.001, and ****P < 0.0001.

To study the effects of prophylactic fluoxetine on infection outcome, we conducted a pretreatment dose titration as shown in Fig. 1A based on previously published chronic dosing regimens (*22*, *23*). Following this pretreatment, we challenged mice intraperitoneally with our polymicrobial sepsis model, which consists of a 1:1 mixture of the Gram-negative bacterium *Escherichia coli* O21:H+ (*24*) and the Gram-positive bacterium *Staphylococcus aureus* subspecies Rosenbach [American Type Culture Collection (ATCC) 12600] that was originally isolated from human pleural fluid (*25*). Fluoxetine pretreatment protected against infection-induced mortality in a dose-dependent manner (Fig. 1B). By contrast, therapeutic administration of fluoxetine to infected mice did not confer protection (fig. S1A). In addition to promoting survival, fluoxetine pretreatment protected from clinical signs of disease including morbidity and hypothermia (Fig. 1, C and D). Given that pretreatment of 40 mg/kg conferred the highest level of protection, we used this dose for our studies.

As sepsis is due to life threatening organ dysfunction, we performed a clinical pathology analysis to understand how fluoxetine pretreatment affects organ damage in our sepsis model. We found that both vehicle and fluoxetine-pretreated infected mice exhibited a comparable elevation in circulating levels of Troponin I, which is a marker of cardiac muscle damage (Fig. 1E). Fluoxetine-pretreated infected mice exhibited reduced circulating levels of other markers of heart damage compared to vehicle-pretreated infected mice including creatine kinase (CK) (which is a marker of both cardiac and skeletal muscle damage) (Fig. 1F), as well as brain natriuretic peptide (BNP), which is released in response to ventricular wall stretch and is a biomarker for heart failure Fig. 1G) (*26*–*29*). Together, these results demonstrate that while fluoxetine- and vehicle-pretreated mice appear to exhibit similar levels of Troponin I, fluoxetine pretreatment protects against ventricular stretch and possibly cardiac failure during sepsis. In addition, we found that fluoxetine-pretreated infected mice were protected from elevated levels of blood urea nitrogen (BUN), which is a marker of kidney damage (Fig. 1H). Last, we found that circulating levels of aspartate transaminase (AST) and alanine transaminase (ALT), which are markers of hepatic damage, were elevated to comparable levels in both vehicle- and fluoxetine-pretreated mice during infection (Fig. 1, I and J). By contrast, fluoxetine pretreatment protects from elevating circulating levels of an additional marker of liver (and bone) damage, alkaline phosphatase (ALP) (Fig. 1K). To better understand whether fluoxetine protects against sepsis-induced liver damage, we conducted a histopathology analysis on livers at 10 hours post-infection. We found that fluoxetine pretreated infected mice exhibited less necrosis, congestion, and hemorrhage, suggesting that fluoxetine pretreatment does indeed confer protection against sepsis-induced liver damage in our model (Fig. 1, L and M, and fig. S1, B to D). Together, these data demonstrate that fluoxetine pretreatment protects from sepsis-induced sickness, multi-organ damage, and death.

### Fluoxetine promotes both pathogen resistance and host-pathogen cooperation

**Fig. 2.**
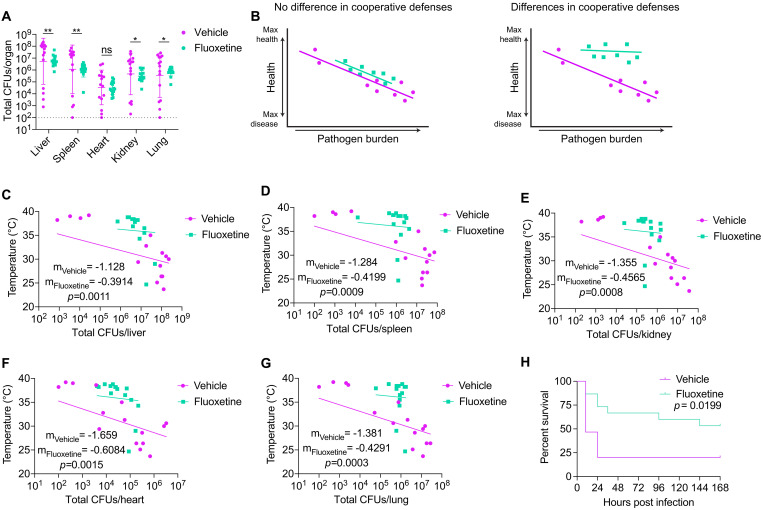
Fluoxetine pretreatment promotes both pathogen resistance and host-pathogen cooperation. (**A**) Total pathogen burden analysis from vehicle- or fluoxetine-pretreated mice at 8 to 10 hours post infection. *n* = 15 per condition, three independent experiments combined. Data represent geometric means ± geometric SD. Unpaired *t* tests. (**B**) Hypothetical reaction norms showing no difference in host-pathogen cooperation (left) and differences in cooperation (right) curves. (**C** to **G**) Reaction norm analyses plotting body temperature at the time of dissection against total CFUs for mice in (A). (C) Liver. (D) Spleen. (E) Kidney. (F) Heart. (G) Lung. Semilog linear regression, *y*-intercept constrained to average of uninfected temperature, Extra sum-of-squares *F* test to compare slopes. Three independent experiments combined. (**H**) Survival of vehicle- or fluoxetine-pretreated mice that were challenged with a heat-killed 1:1 mixture of *S. aureus* and *E. coli*. *n* = 15 per condition, two independent experiments combined. Log-rank analysis. *P < 0.05 and **P < 0.01. ns, not significant.

To determine the contribution of fluoxetine treatment to antagonistic and cooperative defenses, we measured the colony forming units (CFUs) of *Escherichia coli* and *Staphylococcus aureus* in the target organs of the pathogens at 8 hours post-infection. Fluoxetine-pretreated mice had significantly lower total pathogen burdens in their liver, spleen, kidneys, and lungs, but not the heart (Fig. 2A). *E. coli* and *S. aureus* levels followed similar trends in these organs (fig. S1, E and F). These data indicate that fluoxetine pretreatment has antimicrobial effects either via direct actions on the pathogen and/or by heightening the host resistance response to the infection. In agreement with published studies, we found that fluoxetine inhibited *E. coli* and *S. aureus* growth at near clinically relevant concentrations in vitro (fig. S1, G and H) (*30*, *31*), suggesting that fluoxetine’s direct antimicrobial effects may contribute to the observed decrease in pathogen burden.

Because there were differences in pathogen burdens in almost all the target organs analyzed, we needed to use a reaction norm analysis to determine the contributions of fluoxetine treatment to host-pathogen cooperation (*2*, *32*). This involves plotting host health against pathogen burdens and examining how host health changes as pathogen burdens change for each treatment group. A shallower slope indicates a better ability to maintain health over a range of pathogen and therefore to improve adaptation to the infected state (Fig. 2B). We generated reaction norms for each organ using body temperature at 8 hours post-infection as our readout for health and plotted against the pathogen burdens observed for the respective organ at the same time point. We found for all organs that fluoxetine-pretreated infected mice exhibited shallower slopes compared to vehicle-treated infected mice, in addition to the expected shift along the diagonal that indicates differences in pathogen burdens (Fig. 2, C to G, and fig. S1, I to R). Last, to further confirm that fluoxetine has protective effects independent of pathogen burden control, we found that fluoxetine pretreatment conferred significant protection to mice challenged with heat-killed mixture of *S. aureus* and *E. coli* (Fig. 2H). This indicates that fluoxetine pretreatment facilitates host adaptation to the infected state and therefore host-pathogen cooperation via an antivirulence and/or disease tolerance mechanism(s). Together, our data suggest that fluoxetine protects from sepsis-induced disease and mortality via multiple strategies including antimicrobial effects and also by promoting host-pathogen cooperation.

### Fluoxetine-mediated protection from sepsis is independent of peripheral serotonin

**Fig. 3.**
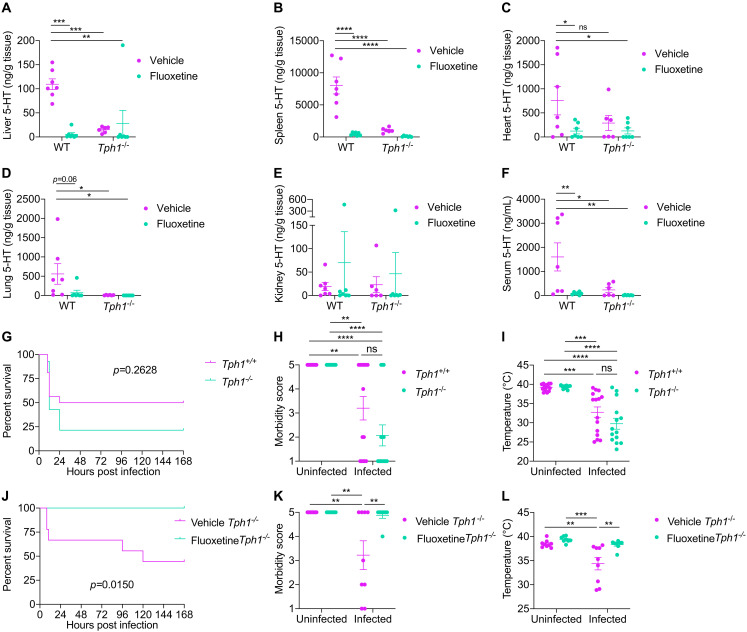
Fluoxetine mediated protection from sepsis is independent of peripheral serotonin. (**A** to **F**) Serotonin (5-HT) levels of wild-type (WT) and *Tph1*^−/−^ mice at 8 to 10 hours post-infection in vehicle- or fluoxetine-treated mice infected with polymicrobial sepsis. (A) Liver. (B) Spleen. (C) Heart. (D) Lung. (E) Kidney. (F) Serum. *n* = 6 to 7 per condition, two independent experiments combined. Two-way ANOVA with Dunnett’s multiple comparisons test against WT vehicle. (**G** to **I**) (G) Survival, (H) most severe morbidity score exhibited over course of infection by each mouse, and (I) minimum temperature exhibited over course of infection by each mouse of *Tph1*^+/+^ or *Tph1*^−/−^ littermates infected with polymicrobial sepsis. *n* = 14 to 16 per condition, four independent experiments combined. For survival, log-rank analysis. For morbidity score and temperature, two-way ANOVA with Tukey’s multiple comparisons. (**J** to **L**) (J) Survival, (K) most severe morbidity score exhibited over course of infection by each mouse, and (L) minimum temperature exhibited over course of infection by each mouse of *Tph1*^−/−^ mice treated with vehicle or fluoxetine infected with polymicrobial sepsis. *n* = 8 to 9 per condition, three independent experiments combined. For survival, log-rank analysis. For morbidity score and temperature, two-way ANOVA with Tukey’s multiple comparisons. In all panels, data represent means ± SEM. *P < 0.05, **P < 0.01, ***P < 0.001, and ****P < 0.0001.

Peripheral serotonin has been reported to contribute to sepsis pathology and mortality in murine models of sepsis (*33*, *34*). Enterochromaffin cells produce most of the peripheral serotonin. In these cells, tryptophan hydroxylase 1 (TPH1) converts tryptophan to 5-hydroxytryptophan (5-HTP), which is then decarboxylated to generate serotonin (5-HT). Serotonin can then be secreted luminally into the gut or basally into circulation contributing to the peripheral serotonin pool (*35*). Once in circulation, serotonin is taken up and sequestered by platelets through the serotonin transporter. Upon activation, platelets release serotonin. SSRIs deplete peripheral serotonin by inhibiting both its export out of enterochromaffin cells and its uptake into platelets (*36*). We measured serotonin levels in infected fluoxetine pretreated wild-type mice and compared to *Tph1^−/−^* mice that genetically lack peripheral serotonin. Fluoxetine pretreated wild-type mice exhibited reduced levels of serotonin in the liver, spleen, lung, and heart comparable to what we observed in these same organs harvested from *Tph1^−/−^* mice (Fig. 3, A to D). Kidney serotonin levels were equivalent across all groups, albeit kidneys had the lowest level of all organs assayed (Fig. 3E). Furthermore, serum serotonin levels were reduced in fluoxetine-pretreated infected mice to comparable levels as we observed in serum from *Tph1^−/−^* mice (Fig. 3F). In addition, at earlier time points throughout polymicrobial sepsis infection, vehicle-pretreated wild-type mice exhibited elevated levels of circulating serotonin, while fluoxetine-pretreated infected wild-type mice were protected from this infection induced increase in circulating serotonin levels (fig. S2, A and B). These differences were independent of platelet activation as measured by thrombocytopenia (fig. S2, C and D). To test the hypothesis that fluoxetine-mediated protection from sepsis was dependent on its actions on peripheral serotonin, we first measured susceptibility of *Tph1^−/−^* mice challenged with polymicrobial sepsis. We found that *Tph1^−/−^* mice were not protected from disease and mortality compared to *Tph1^+/+^* littermates (Fig. 3, G to I). We then tested whether the protective effects of fluoxetine pretreatment were abolished in *Tph1^−/−^* mice. Unexpectedly, we found that 100% of *Tph1^−/−^* mice treated with fluoxetine were protected from sepsis-induced mortality, while ~50% of vehicle-treated infected *Tph1^−/−^* mice succumbed to the challenge (Fig. 3J). Fluoxetine pretreatment also protected *Tph1^−/−^* mice from clinical signs of disease (Fig. 3, K to L). Together, these data demonstrate that in mice lacking peripheral serotonin, fluoxetine still confers protection against sepsis induced morbidity and mortality, suggesting that the fluoxetine-mediated protection during polymicrobial sepsis is independent of peripheral serotonin.

### IL-10 is necessary for fluoxetine-mediated protection from sepsis

**Fig. 4.**
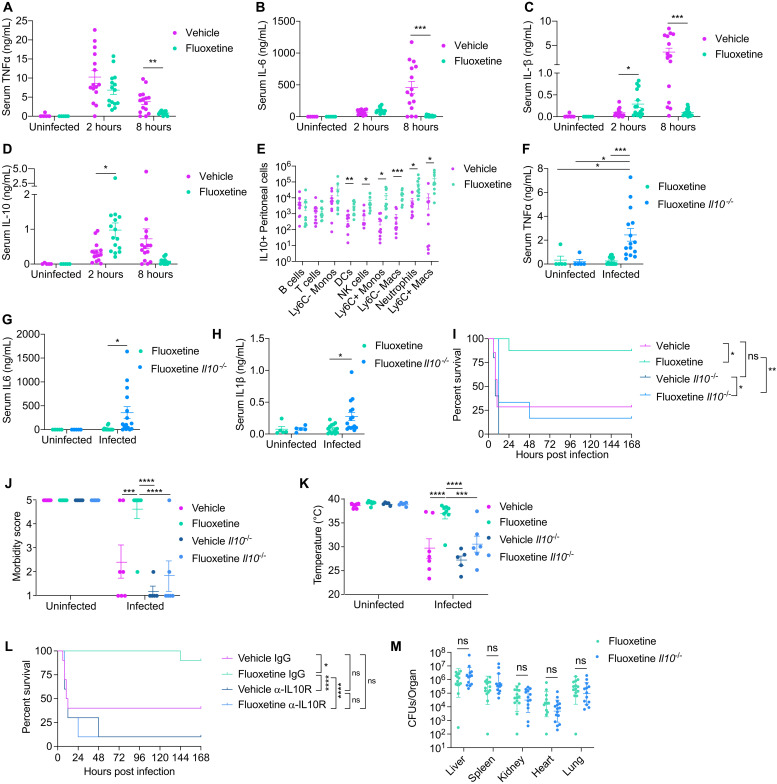
IL-10 is necessary for fluoxetine-mediated protection from sepsis. (**A** to **D**) Circulating levels of (A) TNFα, (B) IL-6, (C) IL-1β, and (D) IL-10 in vehicle- or fluoxetine-pretreated mice infected with polymicrobial sepsis. *n* = 5 to 15 per condition, three independent experiments combined. Data represent means ± SEM. Unpaired *t* tests with Holm-Sidak multiple comparisons correction. (**E**) Flow cytometry analysis of peritoneal lavage cells 2 hours post-infection from vehicle- or fluoxetine-pretreated mice infected with polymicrobial sepsis. *n* = 10 per condition, two independent experiments combined. Data represent means ± SEM. Unpaired *t* tests. (**F** to **H**) Circulating levels of (F) TNFα, (G) IL-6, and (H) IL-1β at 10 hours post-infection in WT or *Il10^−/−^* mice pretreated with fluoxetine infected with polymicrobial sepsis. *n* = 5 to 11 per condition, two independent experiments combined. Data represent means ± SEM. Two-way ANOVA with Tukey’s multiple comparisons test. (**I** to **K**) (I) Survival, (J) most severe morbidity score exhibited over course of infection by each mouse, and (K) minimum temperature exhibited over course of infection by each mouse of WT or *Il10^−/−^* mice treated with vehicle or fluoxetine infected with polymicrobial sepsis. *n* = 5 to 8 per condition, two independent experiments combined. Data represent means ± SEM. For survival, log-rank analysis. For morbidity and temperature, two-way ANOVA with Tukey’s multiple comparison test. (**L**) Survival of infected vehicle- and fluoxetine-pretreated mice treated with an α-IL-10R antibody or isotype control. *n* = 10 mice per condition, two experiments combined. Log-rank analysis. (**M**) Total pathogen burden analysis at 10 hours post-infection from WT or *Il10^−/−^* mice treated with fluoxetine infected with polymicrobial sepsis 10 hours post-infection. *n* = 8 to 14 per condition, two to three independent experiments combined. Data represent geometric means ± geometric SD. Unpaired *t* tests. *P < 0.05, **P < 0.01, ***P < 0.001, and ****P < 0.0001.

Sepsis pathogenesis can be driven by an overexuberant inflammatory response to infection (*7*, *37*, *38*). In vitro and in vivo studies have demonstrated that fluoxetine has anti-inflammatory effects (*39*–*42*). In a rat endotoxemia model, fluoxetine treatment protected from elevated circulating levels of the pro-inflammatory cytokines tumor necrosis factor–α (TNFα), IL-6, and IL-1β (*43*). In our polymicrobial sepsis model, at 2 hours post-infection, we found that fluoxetine-pretreated mice had equivalent induction of TNFα and IL-6 and significantly higher levels of serum IL-1β compared to vehicle-treated infected mice (Fig. 4, A to C). This inflammatory response was resolved in fluoxetine-pretreated mice by 8 hours post-infection, while vehicle-pretreated infected mice exhibited markedly increased the levels of these cytokines at this late time point with a similar magnitude and pattern exhibited by human septic patients (Fig. 4, A to C) (*44*). Hepatic transcript levels of these cytokines showed similar induction and kinetic patterns as we observed in serum (fig. S2, E to G).

Pro-inflammatory cytokine production can be limited by concurrent anti-inflammatory cytokine production (*38*). At 2 hours post-infection, fluoxetine-pretreated mice exhibited significantly greater induction of circulating and hepatic levels of IL-10 compared to vehicle-pretreated mice (Fig. 4D and fig. S2H). Furthermore, we found greater peritoneal recruitment of multiple IL-10^+^ innate immune cell populations, including natural killer cells, neutrophils, Ly6C^+^ monocytes, Ly6C^−^ macrophages, and Ly6C^+^ macrophages, from fluoxetine-pretreated infected mice 2 hours post-infection (Fig. 4E and fig. S2, I to K). However, we did not observe significantly different percentages of IL-10^+^ cells in these populations (fig. S2I), and we did not observe changes in numbers or percentage of IL-10^+^ B cells, T cells, and Ly6C^−^ monocytes (Fig. 4E and fig. S2, I to K). Fluoxetine pretreatment had no effect on the amount of IL10^+^ cells in the liver or spleen during infection (fig. S2, L and M). To test the importance of IL-10 for fluoxetine mediated protection from sepsis, we subjected *Il10^−/−^* mice to our fluoxetine pretreatment paradigm (Fig. 1A) and measured pro-inflammatory cytokine production. The anti-inflammatory effects of fluoxetine at the later stages of infection were abrogated in *Il10^−/−^* mice (Fig. 4, F to H, and fig. S2, N to P). Furthermore, fluoxetine pretreatment did not protect *Il10^−/−^* mice from sepsis-induced morbidity, and protection against mortality was reduced by more than 70% (Fig. 4, I to K). In addition, we found that treatment with an anti-IL-10R (α-IL-10R) antibody abolished the protective effects of fluoxetine on survival (Fig. 4L). By contrast, administration of recombinant IL-10 (rIL-10) using a variety of dosing regimens was not sufficient to protect mice that were not pretreated with fluoxetine from sepsis induced lethality (fig. S2, Q and R). Last, from our pathogen burden analysis, we found that there were no differences in total pathogen burden, nor independently *E. coli* or *S. aureus* burden, between fluoxetine-treated infected wild-type and *Il10^−/−^* mice (Fig. 4M and fig. S3, A and B). Instead, as revealed from our reaction norm analysis, fluoxetine-treated *Il10^−/−^* mice exhibited steeper slopes both when analyzing total CFUs or either *E. coli* or *S. aureus* CFUs individually (fig. S3, C to G). Together, these data demonstrate that IL-10 is necessary for the anti-inflammatory effects of fluoxetine and the protection from sepsis-induced disease and mortality mediated by fluoxetine pretreatment. Last, our data demonstrate that the ability of fluoxetine to promote host adaptation to the infection and cooperative defenses is dependent on IL-10.

### Fluoxetine protects from sepsis-induced hypertriglyceridemia

**Fig. 5.**
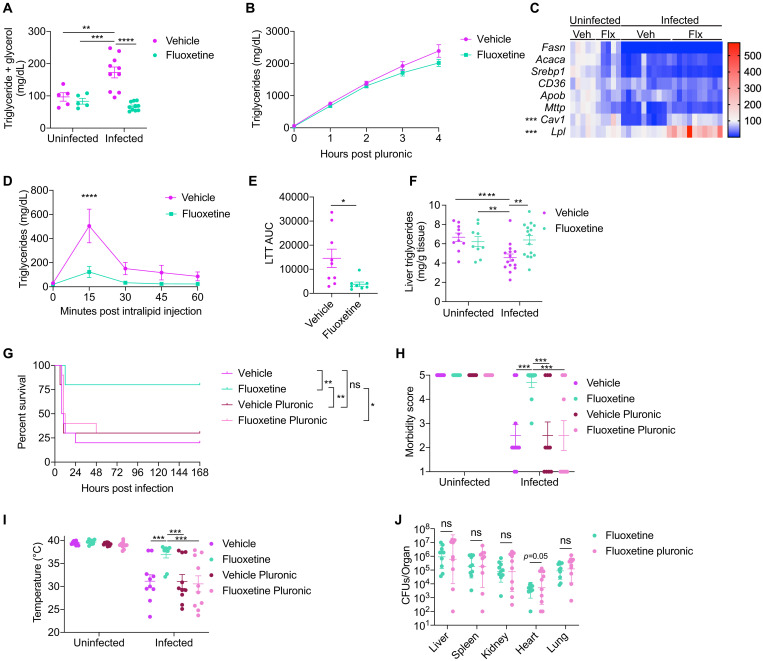
Fluoxetine protects from sepsis-induced dyslipidemia. (**A**) Serum levels of triglycerides + glycerol at 8 to 10 hours post-infection in vehicle- or fluoxetine-infected mice. *n* = 5 to 10 per condition, two independent experiments combined. (**B**) Hepatic triglyceride export in vehicle or fluoxetine infected mice. *n* = 10 per condition, one independent experiment shown. (**C**) Hepatic transcript levels of lipid metabolism-related genes at 8 to 10 hours post-infection. *n* = 5 to 10 per condition, two independent experiments combined. (**D** and **E**) Lipid tolerance test (LTT) at 7 hours post-infection *n* = 8 to 9 per condition, two independent experiments combined. (D) Serum triglyceride levels and (E) area under the curve analysis of (D). One replicate was done in parallel with the LTT performed in Fig. 6 (E and F), and the fluoxetine WT condition was shared between experiments. Therefore, three of the fluoxetine WT mice are also plotted in Fig. 6 (E and F). (**F**) Hepatic triglyceride levels at 8 hours post-infection. *n* = 9 to 15 per condition, three independent experiments combined. (**G** to **I**) (G) Survival, (H) most severe morbidity score exhibited over course of infection by each mouse, and (I) minimum temperature exhibited over course of infection by each mouse ± Pluronic F-127 injection at the time of infection. *n* = 10 per condition, two independent experiments combined. (**J**) Total pathogen burden analysis at 8 to 10 hours post-infection ± Pluronic F-127 injection at the time of infection. *n* = 10 per condition, two independent experiments combined. Data represent ±SEM or geometric means ± geometric SD for CFU analysis. Unpaired *t* test, two-way ANOVA with Tukey’s multiple comparisons, or unpaired *t* tests with Holm-Sidak multiple comparisons correction. *P < 0.05, **P < 0.01, ***P < 0.001, and ****P < 0.0001.

Sepsis causes disturbances in lipid homeostasis including elevated levels of circulating triglycerides (*45*). Fluoxetine usage in patients has been associated with changes in circulating triglycerides and other lipids (*20*). In vitro, fluoxetine has been shown to regulate aspects of lipid homeostasis (*46*). From our ex vivo lipolysis assay, we found that vehicle and fluoxetine pretreated mice had comparable levels of lipolysis in the inguinal white and gonadal adipose tissue (IWAT and GWAT, respectively) during infection (fig. S4, A and B). The analysis of the serum revealed that wild-type mice challenged with our polymicrobial sepsis infection exhibit an increase in circulating triglycerides, while fluoxetine-pretreated mice maintain levels comparable to uninfected mice during infection (Fig. 5A). Triglyceride levels are regulated exogenously by diet and by endogenous mechanisms. As part of our infection protocol, we withhold food for the first 12 hours during polymicrobial sepsis, thus differences in food consumption cannot contribute to differences in triglyceride metabolism. This suggests that endogenous processes are responsible for the observed differences in triglyceride levels between treatment groups. We first tested whether fluoxetine-pretreatment affected hepatic triglyceride export during infection. To test this, we injected vehicle- and fluoxetine-pretreated mice with Pluronic F-127 during infection and measured serum triglyceride levels every hour post-infection for 4 hours. Pluronic F-127 inhibits the activity of lipoprotein lipase (LPL), and so any changes detected in serum triglyceride levels is suggested to reflect hepatic triglyceride export (*47*). Fluoxetine-pretreated infected mice showed a slight but insignificant reduction in triglyceride levels at 4 hours post-Pluronic F-127 injection (Fig. 5B). That said, blocking hepatic triglyceride export with the microsomal triglyceride transport protein (Mttp) inhibitor lomitapide did not significantly influence polymicrobial sepsis survival (fig. S4C). Furthermore, fluoxetine-treated mice exhibited no difference in hepatic transcript levels of fatty acid (FA) transport (*Cd36*), lipogenesis (*Fasn*, *Acaca*, and *Srebp1*), nor triglyceride export-related genes (*Apob* and *Mttp*) (Fig. 5C).

Increased peripheral uptake of triglycerides is an additional endogenous process that will regulate circulating triglyceride levels. We observed fluoxetine-pretreated mice to have increased hepatic transcript levels of *Lpl* and *Cav1*, genes responsible for endocytosis of cholesterol and triglycerides (Fig. 5C). To test whether fluoxetine regulates triglyceride uptake during infection we performed a lipid tolerance test (LTT) by injecting infected mice intravenously with a bolus of intralipid and measuring the clearance of triglycerides from the blood. We found that infected mice that received fluoxetine pretreatment were more lipid tolerant compared to vehicle-treated mice, demonstrating that fluoxetine pretreatment increases peripheral uptake of triglycerides (Fig. 5, D and E). Furthermore, fluoxetine pretreatment protected mice from a drop in hepatic triglyceride levels during infection (Fig. 5F). To determine whether triglyceride uptake is necessary for fluoxetine mediated protection, we administered Pluronic F-127 to mice during infection to inhibit LPL activity and triglyceride uptake and monitored disease progression. We found that Pluronic F-127 treatment abolished the protective effects of fluoxetine pretreatment in polymicrobial sepsis challenged animals (Fig. 5G). Fluoxetine-/pluronic-pretreated infected mice exhibited increased mortality and more severe clinical signs of disease compared to fluoxetine-pretreated infected mice (Fig. 5, H and I). We found that there were no differences in total pathogen burden between fluoxetine and fluoxetine/pluronic mice in the liver, spleen, kidney, and lung, and there was a near significant increase in the hearts of fluoxetine/pluronic mice (Fig. 5J). When we analyzed each pathogen independently, we found that there was no difference in *S. aureus* CFUs between conditions, while there was an increase in *E. coli* CFUs in the livers, kidneys, hearts, and lungs of fluoxetine/pluronic mice (fig. S4, D and E). Our reaction norm analysis demonstrates that fluoxetine/pluronic mice exhibit steeper slopes across all five organs assayed both when analyzing total CFUs and either *E. coli* or *S. aureus* CFUs individually (fig. S4, F to T). Together, these data demonstrate that fluoxetine pretreatment promotes triglyceride uptake during polymicrobial sepsis, which is necessary to promote host-pathogen cooperation, resulting in protection from infection induced morbidity and mortality.

### IL-10 is necessary for fluoxetine-mediated protection from hypertriglyceridemia during sepsis

**Fig. 6.**
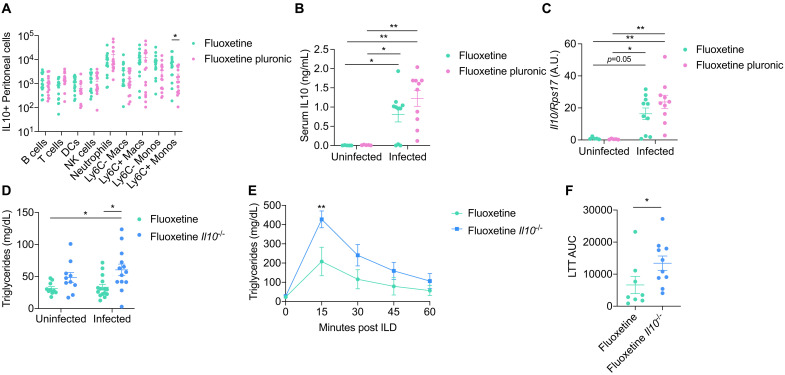
IL-10 is necessary for fluoxetine mediated protection from hypertriglyceridemia during sepsis. (**A** to **C**) (A) Peritoneal lavage flow cytometry, (B) circulating IL-10, and (C) hepatic *Il10* transcription at 2 hours post-infection in fluoxetine-pretreated mice injected with Pluronic F-127 at the time of infection with polymicrobial sepsis. For (A), *n* = 15 per condition, two independent experiments combined, unpaired *t* tests. For (B) and (C), *n* = 5 to 10 mice per condition, one representative experiment shown, two-way ANOVA with Tukey’s multiple comparisons. (**D**) Circulating levels of triglycerides at 8 to 10 hours post-infection in fluoxetine-pretreated WT or *Il10^−/−^* mice infected with polymicrobial sepsis. *n* = 10 to 15 per condition, three independent experiments combined. Two-way ANOVA with Tukey’s multiple comparisons. (**E** and **F**) LTT at 7 hours post-infection in fluoxetine-treated WT or *Il10^−/−^* mice infected with polymicrobial sepsis. *n* = 8 to 10 mice per condition, two independent experiments combined. One replicate was done in parallel with the LTT performed in Fig. 5 (D and E), and the fluoxetine WT condition was shared between experiments. Therefore, three of the fluoxetine WT mice are also plotted in Fig. 5 (D and E). For LTT, two-way ANOVA with Sidak multiple comparisons between vehicle and fluoxetine at each time point. For area under the curve (AUC), unpaired Student’s *t* test. In all panels, data represent means ± SEM. *P < 0.05, **P < 0.01. A.U., arbitrary unit.

We next asked what the relationship is between the fluoxetine-mediated effects on IL-10 and triglycerides. We first tested whether triglyceride uptake is required for fluoxetine-induced IL-10 production. Pluronic decreased the number of IL-10^+^ peritoneal monocytes and decreased the percentage of IL-10^+^ peritoneal monocytes and dendritic cells 2 hours post-infection in fluoxetine-treated mice but did not affect recruitment of IL-10^+^ cells or IL-10 production in any other cell type assayed (Fig. 6A and fig. S4U). Conversely, pluronic did not impact circulating serum levels of IL-10 or hepatic *Il10* transcription 2 hours post-infection in fluoxetine-treated mice (Fig. 6, B and C). Together, these data indicate that some peritoneal cell types require triglyceride uptake to increase IL-10 production during sepsis in fluoxetine-pretreated mice, but ultimately the fluoxetine-mediated increase in circulating and hepatic IL-10 during sepsis is independent of triglyceride uptake.

We next tested whether IL-10 production is required for maintenance of triglyceride levels during sepsis. While we found no differences in the levels of circulating triglycerides when uninfected, fluoxetine-pretreated infected *Il10^−/−^* mice exhibited significantly elevated levels of circulating triglycerides compared to fluoxetine-pretreated infected wild-type mice (Fig. 6D). From our LTT analysis, we found that fluoxetine-pretreated *Il10^−/−^* infected mice were less lipid tolerant compared to fluoxetine-pretreated infected wild-type mice (Fig. 6, E and F), demonstrating that IL-10 is necessary for fluoxetine-mediated increased peripheral uptake of lipids during infection. Together, our data suggest that fluoxetine-mediated effects on lipid uptake and protection from hypertriglyceridemia are dependent on IL-10. By contrast, increased lipid uptake and protection from hypertriglyceridemia is not necessary for the fluoxetine-mediated effects on IL-10 during infection.

### Fluoxetine sustains cardiac glucose oxidation during sepsis

To begin to understand how fluoxetine protects from sepsis-induced disease and mortality, we initiated lines of investigation to understand how fluoxetine regulation of IL-10 and triglycerides protect from organ dysfunction and damage. We chose to focus our efforts on the heart because we found that fluoxetine-treated mice are protected from elevated levels of BNP, suggesting protection from ventricular stretch and possibly heart failure (Fig. 1G). Furthermore, previous research has demonstrated a cardioprotective effect of SSRIs (*48*). Dysregulation in cardiac metabolism is proposed to be a driver of sepsis mortality in humans (*49*). Under physiological conditions, FAs are the main energy substrate for the heart to fuel FA oxidation (FAO), and recent research has suggested that triglyceride utilization by the septic heart in mice is necessary for survival (*50*). Ectopic accumulation of triglycerides can occur in settings of hypertriglyceridemia and when there are deficiencies in FAO (*51*). While we found fluoxetine pretreatment to protect mice from ectopic accumulation of triglycerides in the heart in an IL-10– and LPL-dependent manner (fig. S5, A to C), we found no differences in the levels of active (dephosphorylated) acetyl-CoA carboxylase 1/2 (ACC1/2), a critical regulator of FAO across any treatment groups (fig. S5, D to I). We also found no difference in the expression of genes involved in FAO including *Pgc1a*, *Pgc1b*, *Acadm*, *Erra*, and *Cpt1b* in the hearts of fluoxetine-pretreated and vehicle-pretreated infected mice (fig. S5J). Together, these indirect measurements suggest that fluoxetine pretreatment does not change cardiac FAO during sepsis and that FAO is not impaired in vehicle-pretreated mice that die from sepsis.

**Fig. 7.**
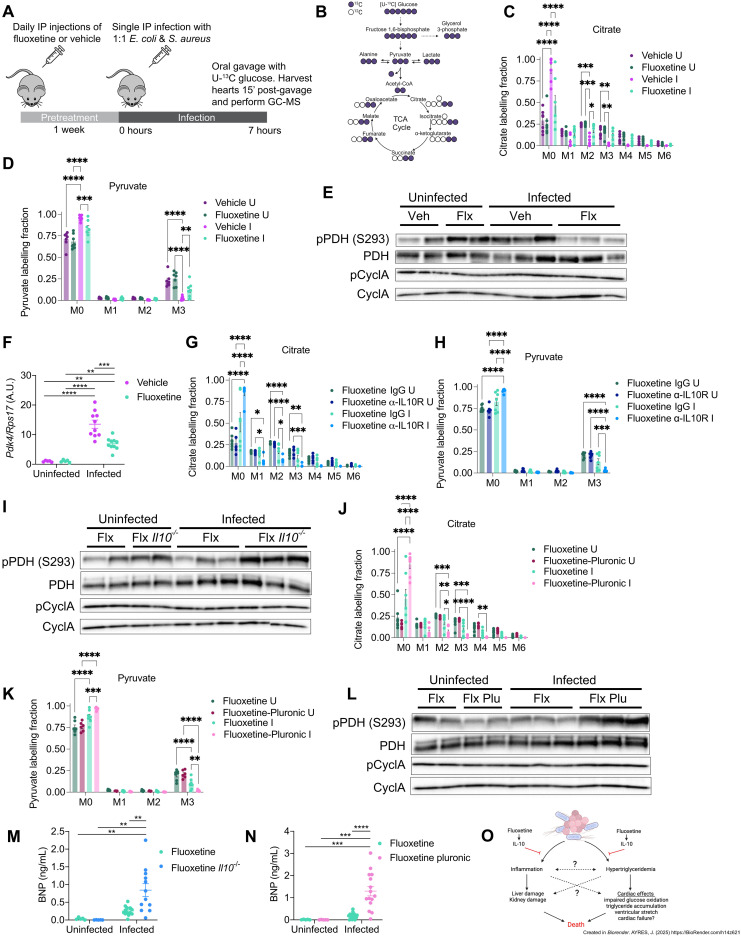
IL-10 and protection from hypertriglyceridemia are necessary for fluoxetine to sustain cardiac glucose oxidation during sepsis. (**A** and **B**) U-^13^C glucose tracing (A) experimental protocol and (B) labeling strategy. (**C** and **D**) U-^13^C glucose labeling of at 7 hours post-infection. (C) Citrate labeling fraction. (D) Pyruvate labeling fraction. *n* = 7 per condition, one experiment shown. (**E**) Western blot of pyruvate dehydrogenase (PDH) complex, phospho-PDH, and cyclophilin A of hearts at 8 to 10 hours post-infection. *n* = 2 to 3 per condition, representative of three independent experiments. (**F**) Cardiac *Pdk4* transcript levels at 10 hours post-infection. *n* = 5 to 10 per condition, two replicates combined. (**G** to **H**) U-^13^C glucose labeling at 7 hours post-infection of hearts from mice injected with isotype control or anti-IL10R. (G) Citrate labeling fraction. (H) Pyruvate labeling fraction. *n* = 7 per condition, one experiment shown. (**I**) Western blot of PDH, phospho-PDH, and cyclophilin A of hearts at 8 to 10 hours post-infection. *n* = 2 to 3 per condition, representative of two independent experiments. Lower right corner of gel ripped after running. The lower right corner and rest of gel were pieced together for transferring. (**J** and **K**) U-^13^C glucose labeling at 7 hours post-infection in hearts from mice injected with pluronic or water. (J) Citrate labeling fraction. (K) Pyruvate labeling fraction. *n* = 6 per condition, one experiment shown. (**L**) Western blot of PDH, phospho-PDH, and cyclophilin A of hearts at 8 to 10 hours post-infection mice injected with water or Pluronic F-127 at the time of infection. *n* = 2 to 3 per condition, representative of two independent experiments. (**M**) Circulating BNP levels at 8 to 10 hours post-infection. *n* = 5 to 15 per condition, three independent experiments combined. (**N**) Circulating BNP levels at 8 to 10 hours post-infection in mice injected with water or Pluronic. *n* = 5 to 15 per condition, three independent experiments combined. (**O**) Model schematic. Two-way ANOVA with Tukey’s multiple comparisons. Uncropped blots are shown in figs. S6 and S7. In all panels, data represent means ± SEM. *P < 0.05, **P < 0.01, ***P < 0.001, and ****P < 0.0001.

While FAs are the main energy source for the heart, glucose utilization is also important for cardiac metabolism under homeostatic conditions and changes in glucose oxidation can occur in the septic heart (*52*). Glucose oxidation is proposed to be of benefit to the heart during sepsis because glucose is a more efficient energy source in terms of oxygen consumption compared to FAs (*53*). Furthermore, ectopic accumulation of lipids can interfere with glucose metabolism, for example, by inhibiting glycolytic enzymes or impairing insulin sensitivity (*54*–*56*). To assess the levels of glucose oxidation in the heart, we performed stable isotope metabolomics using a U-^13^C glucose labeling strategy to profile tricarboxylic acid (TCA) intermediates in the hearts of vehicle- and fluoxetine-pretreated infected and uninfected mice (Fig. 7, A and B). While we found no differences in the relative abundance (fig. S6, A to C), we did find that the levels of labeled TCA intermediates were significantly lower in vehicle-infected mice compared to uninfected controls (Fig. 7C and fig. S6, D and E). By contrast, fluoxetine-infected mice exhibited comparable labeling of TCA intermediates as uninfected controls (Fig. 7C and fig. S6, A to E). Thus, fluoxetine pretreatment protects from sepsis-induced impairment of glucose oxidation in the heart.

To understand why infected vehicle-pretreated mice have reduced glucose flux into the TCA cycle, we looked at regulators of this process including glycolysis, which generates pyruvate from glucose, and pyruvate dehydrogenase (PDH), which regulates the amount of pyruvate that enters the TCA cycle. Vehicle-pretreated infected mice had reduced labeling of pyruvate, in addition to glycolytic intermediates including glycerol 3-phosphate and lactate, while fluoxetine-pretreated infected mice exhibited comparable levels as uninfected controls (Fig. 7D and fig. S6, F to J). Furthermore, vehicle-pretreated infected mice had an increase in the ratio of pPDH:total PDH compared to vehicle-uninfected wild-type mice (Fig. 7E and fig. S6, K to O). By contrast, fluoxetine pretreatment protected against impairment of glycolysis and inhibition of PDH activity in the heart during sepsis (Fig. 7E and fig. S6, F to O). Consistent with this, *Pdk4*, the major kinase responsible for cardiac PDH phosphorylation, was elevated in vehicle-pretreated mice during infection compared to fluoxetine-pretreated mice (Fig. 7F). The effects of fluoxetine pretreatment on circulating BNP levels and glycolytic flux into the TCA cycle were dependent on both IL-10 and Lpl activity (Fig. 7, G to N; fig. S6, R to T; and fig. S7, A to Y). Together, our data suggest that fluoxetine protects from impairment of glucose oxidation in the septic heart and cardiac ectopic lipid accumulation, and this is dependent on IL-10 protection against hypertriglyceridemia (Fig. 7O).

## DISCUSSION

Historically, basic sepsis research has largely focused on characterizing the inflammatory factors driving disease progression, e.g. IL-1β, TNFα, Toll-like receptor 4, etc., with the assumption that blocking these mechanisms of disease in the clinic with either small molecules or monoclonal antibodies will be sufficient to promote survival. Unfortunately, clinical trials based on this approach have been largely unsuccessful. A new perspective for treating sepsis is needed. In addition to characterizing mechanisms of disease pathogenesis, it is critical to identify mechanisms to promote host-pathogen cooperation and develop therapeutic strategies that target these host encoded responses. In the current study, we identified the SSRI fluoxetine as a prophylactic agent that promotes survival in a murine model of polymicrobial sepsis and revealed the immunometabolic mechanisms by which this occurs (Fig. 7O).

Fluoxetine has been previously used as a pharmacological tool to study the role of serotonin during sepsis (*33*). We found that fluoxetine-mediated protection is independent of peripheral serotonin in our sepsis model. In addition, multiple studies have used orthogonal approaches to study the role of peripheral serotonin in sepsis such as *Tph1^−/−^* mice (*33*, *34*), but we also found that these mice are not protected from mortality and death in our model. There are several factors that may explain the discrepancies between our work and these previously published studies. First, previous studies used LPS and cecal ligation and puncture (CLP) models of sepsis, while we used our polymicrobial sepsis model. Second, previous studies used male mice, while we used females for our study. Notably, while work by Rosen *et al.* (*15*) found that fluvoxamine, another SSRI, protects against mortality following LPS or fecal slurry injection by acting through the sigma-1 receptor; the authors did not test the role of serotonin in their model (*15*). Future work is needed to determine in greater detail under, which circumstances peripheral serotonin is pathogenic, and what molecular targets SSRIs act through to mediate their protective effects.

From our pathogen burden analyses, we found that the protective effects of fluoxetine pretreatment were associated with a reduction in pathogen burdens in the liver, spleen, kidney, and lungs, but not the heart. In agreement with published studies, we found that fluoxetine inhibited *E. coli* and *S. aureus* growth in vitro at near clinically relevant concentrations (*30*, *31*), suggesting that fluoxetine’s direct antimicrobial effects may contribute to the observed decrease in pathogen burden in our model. When pathogen burdens are changing, it can be difficult to reveal pathogen burden–independent effects, and so we performed reaction norm analyses with our dataset. This is a widely accepted analysis in multiple fields as a way to determine the dependency of a host phenotype on pathogen burdens (*2*). With this approach, we revealed fluoxetine-mediated protective effects that are independent of pathogen burdens. To provide additional evidence that fluoxetine has protective effects independent of its antimicrobial effects, we tested whether fluoxetine pretreatment was protective in a nonreplicative sepsis model. Fluoxetine pretreatment protected mice challenged with a heat-killed mixture of our polymicrobial sepsis model from lethality. Together, our data show that fluoxetine has protective effects that are independent of its antimicrobial effects.

Previous reports have suggested that fluoxetine has anti-inflammatory effects (*17*, *18*). In the current study, we found that fluoxetine does not completely abolish the pro-inflammatory response. Instead, fluoxetine controls the degree and duration of the pro-inflammatory response. In fluoxetine pretreated mice, we found that, at the early stages of infection, there is a comparable to higher level of induction of pro-inflammatory cytokines as vehicle-pretreated mice. In parallel, fluoxetine pretreated mice exhibit a greater induction of the anti-inflammatory cytokine IL-10. However, by the later stages of infection, the inflammatory response has returned to baseline in fluoxetine-treated mice, while it persists in vehicle-pretreated mice. Using whole-body *Il10^−/−^* mice, we found that the protective effects of fluoxetine pretreatment against sepsis were largely abolished. As global knockout of IL-10 will have many off target effects that are not directly related to fluoxetine, we also used an α-IL-10R antibody and found that blocking IL-10 signaling during infection did not result in any significant differences in susceptibility of vehicle-pretreated mice to sepsis but did abolish the protective effects of fluoxetine, similar to what we observed with *Il10^−/−^* mice. We did not find the administration of rIL-10 to have any protective effect on mice infected with our polymicrobial sepsis model. This can indicate that IL-10 is necessary for the protective effects mediated by prophylactic fluoxetine but not sufficient for protection. Alternatively, it may indicate that our dosing was not optimized to reveal a protective effect for the use of rIL-10 as a therapeutic.

An outstanding question is: How does fluoxetine increase circulating levels of IL-10 during polymicrobial sepsis? Yeung *et al.* (*57*) recently reported that most of the IL-10 in response to LPS challenge is produced by CD169^+^ macrophages in the spleen. We did not find any differences in IL-10^+^ immune cells in vehicle and fluoxetine pretreated mice during polymicrobial sepsis infection in the spleen. Instead, we found that fluoxetine pretreatment resulted in greater peritoneal recruitment of several different IL-10^+^ innate cell types. In addition, we found increased expression of *Il10* in the liver of fluoxetine-pretreated septic mice; however, we did not find an increase in IL-10^+^ immune cells in the liver, suggesting that an alternative cellular source, such as hepatocytes, may be a relevant source. Future studies are needed to determine the mechanism by which fluoxetine regulates IL-10 and determine the contribution of each of these cell types in fluoxetine’s anti-inflammatory effects on regulating lipid homeostasis and sepsis disease progression.

Sepsis is largely driven by an overexuberant inflammatory response to an infection. While the ability of fluoxetine to regulate the degree and the duration of the pro-inflammatory response likely contributes to its protective effects against sepsis, in the current study, we revealed that a component of fluoxetine’s protective effects involves its ability to increase peripheral lipid uptake and protect from hypertriglyceridemia in an IL-10 dependent manner. We currently do not know the mechanism by which IL-10 protects from hypertriglyceridemia, but it is reasonable to speculate that it may involve that the anti-inflammatory effects of IL-10 as pro-inflammatory cytokines including TNFα and IL-6 can lead to hypertriglyceridemia via their effects on triglyceride physiology (*58*). For example, IL-6 has been shown to influence hepatic lipid accumulation (*59*), and previous work has demonstrated that fluoxetine can drive hepatic triglyceride accumulation in non-sepsis contexts (*60*). In agreement with this, we found that there is increased expression of genes involved in lipid uptake including *Lpl* and *Cav1*, in the liver of fluoxetine-treated septic mice, suggesting that fluoxetine and IL-10 may promote normolipidemia by increasing hepatic triglyceride uptake. When we looked at hepatic triglyceride content, we found that vehicle-pretreated mice had reduced liver triglyceride levels during infection, while fluoxetine-pretreated mice maintain higher levels comparable to uninfected control mice, suggesting that processes downstream of triglyceride uptake may also be enhanced in fluoxetine-pretreated mice. Triglyceride flux analyses should be done in future studies to understand the potential role of the liver in the protection from fluoxetine-mediated protection against sepsis-induced hypertriglyceridemia. In a previous study using a CLP model, Van Wyngene *et al.* (*61*) showed that there is lipid accumulation in the liver during sepsis leading to lipotoxicity as indicated by elevated levels of AST and ALT. In our study, we also found that fluoxetine-pretreated mice exhibit elevated AST and ALT levels comparable to vehicle-pretreated mice, but they are protected from liver damage as indicated by our histopathology analysis and lower ALP levels. These results suggest that the hepatic lipotoxicity reflected in elevated AST and ALT levels is distinct from inflammation-driven histopathological differences and elevated ALP levels. Future research should further investigate the distinct contributions of metabolic and immune derangements to different types of hepatic damage and ultimately the consequences of those injuries on morbidity in sepsis.

Increased circulating triglyceride levels can lead to ectopic accumulation of lipids in the heart and development of cardiovascular disease (*62*). Consistent with this, we found that the elevated circulating triglycerides found in vehicle-treated septic mice were associated with cardiac triglyceride accumulation. Lipid accumulation in the heart can disrupt the metabolic flexibility of the heart (*63*). Based on metabolic flux analyses and indirect measurements, we found that vehicle-pretreated infected mice had reduced levels glucose oxidation in the heart, and this was associated with reduced levels of glycolysis and PDH inhibition. Fluoxetine pretreatment sustained levels of cardiac glycolysis and protected from PDH inhibition during sepsis, maintaining glycolytic flux into the TCA cycle and protecting from sepsis-induced impairment of cardiac glucose oxidation. In vehicle-pretreated sepsis mice, we found that there is an increased expression of cardiac *Pdk4*, a negative regulator of PDH. Increased *Pdk4* expression is often associated with increased FAO (*64*); however, we do not observe changes in FAO in our model. One possibility to explain our observation is that when there is reduced glycolysis and increased triglyceride accumulation, this can lead to greater *Pdk4* expression to facilitate a shift to greater FAO, but when septic, the metabolic flexibility of the heart is impaired and is unable to make this shift. While cardiac triglyceride utilization and FAO have been proposed to be necessary for survival of sepsis (*50*), based on our indirect measurements, we did not find sepsis or fluoxetine to have any effects on cardiac FAO. We propose that maintenance of glucose oxidation in the heart is necessary for protection from septic-induced ventricular stretch and possibly cardiac failure and contributes to fluoxetine’s protective effects on sepsis-induced morbidity and mortality. This is in line with previous work that has demonstrated fluoxetine protects against cardiac damage following aortic constriction (*48*).

A previous study using a mouse model of endotoxemia proposed that GDF15-mediated hepatic export of triglycerides was necessary to support the heart during disease (*50*). In our study, we did not find blocking hepatic triglyceride export with the Mttp inhibitor lomitapide to significantly influence survival, and we did not find differences in FAO in hearts of dying or surviving mice based on indirect measurements. However, there are important differences between our study and the study reported by Luan *et al*. (*50*) that may help explain these discrepancies. First, in our study, fluoxetine-treated mice exhibit elevated serum Troponin during sepsis, while mice with GDF15 are spared from this. This suggests that fluoxetine-mediated effects on the heart are likely different from those in GDF15 mice. Second, the authors investigated GDF15-regulated triglycerides using an LPS model of sepsis, while we used a co-infection model with live bacteria. Third, the Luan *et al.* study used male mice, while we used female mice for our studies. Fourth, Luan *et al*. (*50*) took a different approach to perturb hepatic triglyceride export, which may also explain differences. We used a direct pharmacological approach to inhibit microsomal triglyceride transfer protein. Luan *et al*. (*50*) used a global β-adrenergic receptor knockout. While these mice do exhibit blunted hepatic triglyceride export, they also have been shown to have many other differences that will be relevant for sepsis pathogenesis including cardiac function (*65*–*68*). Last, potential microbiota differences may possibly contribute to differences. Future work should be undertaken to further explore triglyceride metabolism dynamics during sepsis, and how this relates to cardiac damage and function, and whether these findings are clinically applicable to human patients. An important question that arises when considering our work and Luan *et al*. (*50*): Are triglycerides beneficial or detrimental for sepsis? We propose that they should not be viewed as one or the other, but rather their levels need to be kept within a critical range. If triglyceride levels reach below a certain threshold, then increasing them would be beneficial. If triglycerides exceed a threshold, then this can be maladaptive and contribute to sepsis pathogenesis.

Our goal for the current study was to understand how prophylactic use of fluoxetine can protect from sepsis and determine the relationship between fluoxetine’s effects on IL-10 and triglycerides. To determine the dose to use for our studies, we performed a pretreatment dose titration and show the amount normally used in mouse studies (10 mg/kg) to be protective; however, we moved forward with most effective dose for our mechanistic studies (40 mg/kg). Considering clinical studies have shown that fluoxetine is “minimally toxic in doses up to 1500 mg” (*69*), and the half-life of fluoxetine is much shorter in rodents compared to humans (5 to 6 hours for rodents and 1 to 3 days for humans), our findings are likely relevant to the higher end of human dosing strategies (*70*, *71*). While our study focused on prophylactic use of fluoxetine, the mechanistic insights we revealed from our study may reveal potential therapeutic strategies for sepsis. Furthermore, while we did not find that fluoxetine administered during infection to be protective, we only used one dosing regimen, and it is possible that testing of additional dosing strategies may reveal the therapeutic potential of fluoxetine for sepsis.

Our study has revealed how a widely available and safe drug, fluoxetine, promotes immunometabolic host-pathogen cooperation to protect from sepsis induced morbidity and mortality. We currently do not know whether other SSRIs will have similar effects. Depending on the “off-target” effect of fluoxetine, it is possible that different subsets of SSRIs may also be protective. For example, if fluoxetine’s protective effects are mediated through the sigma-1 receptor, then we would hypothesize that fluvoxamine (Ki, 36 nM) and sertraline (Ki, 57 nM) may be even more potent that fluoxetine (Ki, 240 nM) (*72*). By contrast, if the protective effects are mediated through TrkB, then we would speculate that tricyclic antidepressants such as imipramine (Ki, 1.03 μM) and esketamine (Ki, 2.86 μM) would have similar protective effects to fluoxetine (Ki, 1.69 μM) (*73*). Sigma-1 receptor agonists have been previously shown to suppress antitumor immunity in an IL-10–dependent manner (*74*), and TrkB signaling has been shown to reduce inflammation in a spinal cord injury model (*75*); thus, it is reasonable to speculate that fluoxetine may be operating through either receptor in our sepsis model. In addition, it is important to consider the distinct drug metabolism of fluoxetine. Fluoxetine is unique as an SSRI in that it has a very long half-life of 4 to 6 days, and its primary metabolite, norfluoxetine, also has a long half-life with a high affinity for the serotonin reuptake pump (*76*). If the protective effects of fluoxetine are mediated through a mechanism that requires high levels of circulating drug, then we would expect fluoxetine to excel in this protection over other, more quickly metabolized SSRIs.

A recent estimate determined the average cost of the research and development required to bring a new therapy to market as more than $1.5 billion. One of the most cost-effective and quickest strategies to develop treatment strategies is repurposing already approved drugs for new conditions. Our study provides sufficient rationale to further explore the therapeutic uses of SSRIs during infection and to reveal new and exciting immunometabolic targets during sepsis.

### Limitations of study

We found that fluoxetine protects from elevated levels of circulating BNP in our sepsis model. While BNP is used as a biomarker for cardiac failure, an ultrasound was not performed in our studies to validate whether cardiac function was impaired in our model. Similarly, while we did not find that fluoxetine administered during infection to be protective, we only used one dosing regimen, and it is possible that testing of additional dosing strategies may reveal the therapeutic potential of fluoxetine for sepsis. To assess levels of FAO, we relied on indirect measurements rather than direct approaches such as metabolic tracing for flux analyses. Last, we relied on levels of circulating markers to characterize organ damage/dysfunction and identify events that contribute to death and did not perform necropsies to determine the ultimate cause of death.

## MATERIALS AND METHODS

Key resources for this study are listed in Table 1. All primers used in this study are listed in table S1.

**Table 1.** Key resource table. List of key resources used in this study.

REAGENT or RESOURCE	SOURCE	IDENTIFIER
**Antibodies**		
Anti-mouse CD45 FITC (clone I3/2.3)	Biolegend	147709
Anti-mouse CD11b BV785 (clone M1/70)	Biolegend	101243
Anti-mouse CD11c PE/Cy7 (clone N418)	Biolegend	117318
Anti-mouse Ly6G BV421 (clone 1A8)	Biolegend	127627
Anti-mouse Ly6C PerCP/Cy5.5 (clone HK1.4)	Biolegend	128012
Anti-mouse F4/80 PE (clone BM8)	Biolegend	123110
Anti-mouse MHCII BV510 (clone M5/114.15.2)	Biolegend	107635
Anti-mouse NK1.1 APC/Cy7 (clone PK136)	Biolegend	108724
Anti-mouse CD3 PE/CF594 (clone 145-2C11)	Biolegend	562332
Anti-mouse B220 BV711 (clone RA3-6B2)	Biolegend	103255
Anti-mouse CD41 PacBlue (clone MWReg30)	Biolegend	133931
Anti-mouse IL-10 APC (clone JES5-16E3)	eBioscience	17–7101
Anti-mouse CD16/CD32 (Fc Shield) (clone 2.4G2)	Tonbo Biosciences	70–0161-U500
Zombie-UV	Biolegend	423107
Anti-mouse TNFα (clone 1F3F3D4)	eBioscience	14–7325
Biotin-conjugated anti-mouse TNFα (clone XT3/XT22)	eBioscience	13–7326
Anti-mouse IL-6 (clone MP5-20F3)	eBioscience	14–7061
Biotin-conjugated anti-mouse IL-6 (clone MP5-32C11)	eBioscience	13–7062
Anti-mouse IL-1β (clone B122)	eBioscience	14–7012
Biotin-conjugated anti-mouse IL-1β polyclonal	eBioscience	13–7112
Anti-mouse IL-10 (clone JES052A5)	R&D Systems	MAB417
Biotin-conjugated anti-mouse IL-10 polyclonal	R&D Systems	BAF417
Streptavidin HRP	BD Pharmingen	554066
Rabbit anti-mouse ACC (clone C83B10)	Cell Signaling	3676
Rabbit anti-mouse phospho-ACC (Ser79) (clone D7D11)	Cell Signaling	11818
Rabbit anti-mouse PDH (clone C54G1)	Cell Signaling	3205
Rabbit anti-mouse phospho-PDH (Ser293) polyclonal	Cell Signaling	31866
Rabbit anti-mouse Cyclophilin A	Cell Signaling	51418S
Anti-rabbit IgG HRP-linked Ab	Cell Signaling	7074S
Ultra-LEAF purified IgG1 isotype control antibody	BioLegend	400457
Ultra-LEAF purified anti-mouse IL10R	BioLegend	112711
**Chemicals and reagents**		
Fluoxetine hydrochloride	Santa Cruz Biotechnology	sc-201125B
DNase I	Roche	10104159001
RPMI + GlutaMAX	Gibco	61870–036
Penicillin–Streptomycin Solution (100X)	Gen Clone	25–512
HEPES (1 M)	Sigma	H3537
Sodium Pyruvate (100 mM)	Gibco	11360–070
MEM Non-Essential Amino Acids (100X)	Gibco	11140–050
Fetal Bovine Serum	Gibco	10437–028
Brefeldin A (1000X)	eBioscience	00–4506-51
Percoll	Cytiva	17089101
Ack Lysing Buffer	Gibco	A10492–01
Brilliant Stain Buffer	BD Biosciences	566349
Formaldehyde 16%	Fisher	NC9658705
Saponin permeabilization buffer	Invitrogen	00–8333-56
CountBright Plus Absolute Counting Beads	Invitrogen	C36995
Citric acid	Sigma	C2404-100G
Sodium citrate	Fisher Scientific	S279–10
Dextrose	Fisher Scientific	BP350–1
Bacto Yeast Extract	Gibco	212750
Bacto Tryptone	Gibco	211705
Sodium Chloride	Fisher Scientific	S271–3
Difco Agar	BD Biosciences	281210
Eosin Methylene Blue Agar (Levine)	Oxoid	CM0069
Ampicillin	Fisher Scientific	BP1760–25
Neomycin Sulfate	Thermo Scientific	1405-10-3
Vancomycin Hydrochloride	VWR	0990
Metronidazole	Alfa Aesar	H60258
Triton X-100	Promega	H5142
Nonidet P-40	Sigma	492018
Sodium Deoxycholate	Sigma	D6750-100G
Sodium Dodecyl Sulfate	Thermo Scientific	28365
Tris	Millipore Sigma	P1379
Bovine Serum Albumin	Thermo Scientific	BP1600–100
Halt Protease and Phosphatase inhibitor cocktail (100x)	Thermo Scientific	78440
Nupage Tris-Acetate SDS Running Buffer	Thermo Scientific	LA0041
Nupage 7% Tris-Acetate Gel 1.0 mm x 12 well	Thermo Scientific	EA03552BOX
Nupage LDS Sample Buffer (4x)	Invitrogen	NP0007
Protease Inhibitor Cocktail (100x)	Sigma	P8340-5ML
OneBlock Western-CL Blocking Buffer	Prometheus	20–313
β-mercaptoethanol	Sigma	M3148
Pluronic F-127	Sigma	P2443
AllPrep DNA/RNA Mini Kit	Qiagen	80204
RNase-free DNase set	Qiagen	79254
RNase Out	Invitrogen	10777019
SuperScript IV	Invitrogen	18090010
SYBR Green	Biorad	1725125
Intralipid	Sigma	I141-100ML
Lomitapide	Fisher Scientific	L0298100MG
DMSO	Fisher Scientific	BP231–100
Corn oil	Fisher Scientific	AC405435000
rIL-10	Peprotech	210–10
**Critical commercial assays**		
Serotonin ELISA kit	Abcam	ab133053
Mouse cardiac troponin I ELISA kit	Life Diagnostics	NC1052736
Mouse brain natriuretic peptide (BNP) ELISA kit	Cusabio	CSB-E07971m
Lipid Extraction Kit (Chloroform Free)	Abcam	ab211044
L-Type Triglyceride M Enzyme Color A (R1)	FUJIFILM	996–02895
L-Type Triglyceride M Enzyme Color B (R2)	FUJIFILM	998–02992
Multi-Calibrator Lipid	FUJIFILM	464–01601
Pierce BCA Protein Assay Kit	Thermo Scientific	23225
o-phenylenediamine dihydrochloride	Sigma	P3804-50TAB
ProSignal Dura	Prometheus	20–301
ProSignal Femto	Prometheus	20–302
**Oligonucleotides**		
Primers for qPCR	Table S1	N/A
**Mouse models**		
C57BL/6 J	The Jackson Laboratory	000664
Tph1^tm1Bdr^ (*Tph1^−/−^*)	Originally generated by Michael Bader (MDC, Germany), gifted by Laura L Hernandez (UW Madison)	MGI: 2450301
B6.129P2-*Il10^tm1Cgn^*/J (*Il10^−/−^*)	The Jackson Laboratory	002251
**Software**		
SoftMax Pro 5.4.5 Microplate Data Acquisition and Analysis Software	Molecular devices	SMP5ACAD
QuantStudio5 Design and Analysis software v1.5.0	Applied Biosystems	A28140
Image Lab Software 5.2.1	BioRad	12012931
FACSDiva 8.0	BD Biosciences	23–14523-00
FlowJo 10.8.1	FlowJo, LLC	https://www.flowjo.com/
**Other**		
Portable Scale	Ohaus	HH320
Microplate reader	Molecular devices	VERSAMAX
BeadMill24 benchtop bead-based tissue homogenizer	Fischer Scientific	15–340-163
QuantStudio5 Real-Time 384-well	Applied Biosystems	A28140
Gel doc XR + Gel documentation System	BioRad	1708195
NanoDrop One/OneC Microvolume UV–Vis	ThermoFisher Scientific	ND-ONE-W
Microvette Capillary Blood Collection Tubes	Sarstedt	16.440.100
Trans-Blot Turbo Transfer System	Bio-Rad	1704150
LSR II	BD Biosciences	33300478
FACSCanto II	BD Biosciences	338962

### Mice

Female mice of 10 to 12 weeks of age purchased from the Jackson Laboratory or bred in our Association for Assessment and Accreditation of Laboratory Animal Care–certified vivarium were used for the studies described. For experiments with C57BL/6 mice, animals were purchased from the Jackson Laboratory and acclimated in our facility for at least a week before experimentation. *Tph1^−/−^* mice (Tph1^tm1Bdr^, MGI:2450301) (*77*) were a generous gift from L. L. Hernandez (University of Wisconsin-Madison) and bred in house. *Tph1^−/−^* mice were crossed to C57BL/6 mice ordered from the Jackson Laboratory to generate *Tph1^+/−^* mice. *Tph1^+/−^* were then crossed to each other, and *Tph1^−/−^* and *Tph1^+/+^* littermate females were used for experiments. B6.129P2-*Il10^tm1Cgn^*/J mice were purchased from the Jackson Laboratory and bred in house (strain #: 002251). Mice were specific pathogen–free, maintained under a 12-hour light/12-hour dark cycle, and given standard chow diet ad libitum before infection. All animal experiments were done in accordance with The Salk Institute Animal Care and Use Committee approval 12-00038. In accordance with our Institutional Animal Care and Use Committee guidelines, in an effort to reduce the number of animals used for our experiments, where possible and appropriate, experiments were done in parallel and shared controls were used. This is indicated in the figure legends where relevant.

### Bacteria

*E. coli* O21:H+ (*24*) and *Staphylcoccus aureus* (ATCC strain 12600) were used.

### Culturing *E. coli* O21:H+ and *S. aureus* for mouse infection

*E. coli* O21:H+ was incubated on an eosin methylene blue (EMB) plate containing ampicillin sodium salt (1 mg/ml), vancomycin hydrochloride (0.5 mg/ml), neomycin sulfate (1 mg/ml), and metronidazole (1 mg/ml) antibiotics overnight at 37°C to grow single colonies. *S. aureus* was incubated on an LB plate without antibiotics overnight at 37°C to grow single colonies. The next day, a single colony of *E. coli* O21:H+ was inoculated into 100-ml LB-AVNM (using antibiotic concentrations noted above) media, while a single colony of *S. aureus* was inoculated into 5-ml LB without antibiotics. Both cultures were shaken overnight at 37°C (250 rpm). The following morning, the optical density at 600 nm (OD_600_) was measured, and an inoculum with a 1:1 mixture of the bacteria was prepped with the indicated doses in sterile 1x phosphate-buffered saline (PBS) that was used directly for mouse infections.

### Fluoxetine treatment

Fluoxetine hydrochloride powder (Santa Cruz Biotechnology) was suspended in 1x PBS at a concentration of 2 mg/ml. For prophylactic treatment, mice were administered daily intraperitoneal injections of fluoxetine (10, 20, or 40 mg/kg) for 7 days pre-infection. 1x PBS was used as a vehicle control. For therapeutic administration, mice were infected as described above and then injected intraperitoneally with fluoxetine or vehicle (40 mg/kg) at 30 min post-infection.

### Mouse infection models

Mice were infected intraperitoneally with 2 × 10^8^ total bacteria in 500 μl delivered through a 25-gauge needle between ZT0 and ZT2 (6:00 a.m. to 8:00 a.m.). Inoculums were serially diluted and plated to confirm the infectious doses. Immediately after infection, mice were transferred to a fresh cage and food was removed for the first 10 to 12 hours post-infection to control for any potential variations in the sickness-induced anorexic response. Food was also withheld from uninfected controls for the same 10 to 12 hours. Mice were clinically monitored as described below every 2 hours post-infection. Mice that reached clinical end points were euthanized according to our animal protocol. For heat-killed challenge experiments, inoculums containing *S. aureus* (5 × 10^9^ CFU/ml) and *E. coli* (5 × 10^9^ CFU/ml) for a total of 1 × 10^10^ CFU/ml were prepared, incubated at 65°C for 1 hour to kill bacteria. Mice received a 500-μl intraperitoneal challenge of the heat-killed inoculums. Inoculums are plated to confirm no growth.

### Survival

Mice were clinically monitored as described below every 2 hours post-infection. For some experiments, mice were clinically monitored every 2 hours for the first 10 to 12 hours post-infection and then again at 24 hours. Mice that had to be euthanized because they reached clinical end points during the infection, in addition to those that succumb to the infection, were included in our survival analyses.

### Rectal temperature

Rectal temperatures were taken every 2 hours post-infection for the first 10 to 12 hours and then every 24 hours as noted using the Digisense Type J/K/T thermocouple meter.

### Grading system for monitoring morbidity

We use the following morbidity scale to quantify the morbidity of mice. Infected mice are clinically assessed using this morbidity scale every 2 hours post-infection. For some experiments, mice were clinically monitored every 2 hours for the first 10 to 12 hours post-infection and then again at 24 hours.

5. Normal. Normal exploratory behavior, rearing on hind limbs, and grooming.

4. Mild. Reduced exploratory behavior, rearing on hind limbs, and grooming. Slower and/or less steady gait but free ambulation throughout the cage.

3. Moderate. Limited voluntary movement. Slow, unsteady gait for >5 s.

2. Severe. No voluntary movement, but mouse can generate slow, unsteady gait for <5 s.

1. Moribund. Mouse does not move away from stimulation by researcher and cannot right itself.

### IL-10R and rIL-10 treatment

For α-IL-10R experiments, vehicle- and fluoxetine-pretreated mice were injected intraperitoneally with 100 μg of α-IL-10R or isotype control at 24 hours and 2 hours before infection. Survival was monitored. For rIL-10 experiments, mice were infected with polymicrobial sepsis as described above and dosed with vehicle or rIL-10 intraperitoneally as follows: 1 μg at 30 min, 2 and 8 hours post-infection, 1 μg at the time of infection, 1 μg at 4 hours post-infection, or 0.5 μg every hour post-infection for the first 10 hours.

### Ex vivo lipolysis assay

We performed ex vivo lipolysis assays as described in (*78*). Briefly, ~0.025 g of adipose tissue was removed from both the IWAT and GWAT deposits in duplicate. The tissue was incubated in ice-cold 1x PBS during harvest. They were then transferred to 300 μl of Krebs-Ringer bicarbonate Hepes buffer [120 mM NaCl, 4 mM KH_2_PO_4_, 1 mM MgSO_4_, 0.75 mM CaCl_2_, 30 mM Hepes, 10 mM NaHCO_3_, 2% FA-free bovine serum albumin (BSA), and 5 mM glucose] and incubated at 37°C and 5% CO_2_ for 4 hours. The supernatant was collected and frozen for downstream quantification of free FAs and glycerol using the FuijiFilm Wako Chemical’s NEFA assay kit according to the manufacturer’s protocol and the Sigma Free Glycerol Reagent according to the manufacturer’s protocol.

### Histology

Livers were harvested and fixed in 10% neutral-buffered formalin. Then, samples were routinely processed, paraffin-embedded, sectioned at 4 to 5 μm, and hematoxylin and eosin–stained. Tissues were evaluated by a board-certified veterinary pathologist who was blinded to experimental manipulation and scored semi-quantitatively for the following parameters: liver necrosis, liver congestion, and hemorrhage. These parameters were scored on a scale of 0 to 4 with 0 representing normal tissue; 1 represented minimal changes; 2 representing mild changes; 3 representing moderate changes; and 4 representing severe changes relative to a score of 1. Representative images were obtained from glass slides using NIS-Elements BR 3.2 64-bit and plated in Adobe Photoshop. Image white balance, lighting, and/or contrast was adjusted using corrections applied to the entire image.

### Cardiac Troponin I and BNP quantification

Serum from mice was harvested by cardiac puncture. Troponin I levels were measured using an ultra-sensitive mouse cardiac Troponin I enzyme-linked immunosorbent assay (ELISA) kit (Life Diagnostics) according to the manufacturer’s protocol. BNP levels were measured using (BNP Kit). Plates were read on a VersaMax microplate reader manufactured by Molecular Devices, and data analysis was done using SoftMax Pro.

### BUN, CK, ALP, AST, ALT, and quantification

Serums harvested by cardiac puncture were analyzed by IDEXX Bioanalytics.

### Serum triglyceride quantification

As described in the Wako Fujifilm L-Type Triglyceride M Kit protocol, 4 μl of serum from tail bleed (for LTTs or pluronic tests) or cardiac puncture (for circulating levels during infection) was added to a 96-well plate. Standards were generated by using Wako multicalibrator lipids in the following series (mg/dl): 0, 6, 12, 24, 48, 96, 192, and 384. To each well, 90 μl of R1 was added, and the plate was incubated at 37°C for 5 min. The plate was read at 600 and 700 nm. After reading, 30 μl of R2 was added to each well, and the plate was incubated at 37°C for 5 min. The plate was read again at 600 and 700 nm and analyzed following the manufacturer’s instructions. Readings were taken using a 96-well VersaMax microplate reader and SoftMax Pro software. In Fig. 5A, triglyceride measurements were done by IDEXX Laboratories. The protocol used by IDEXX Laboratories does not involve an initial glycerol extraction step before quantification of triglycerides, and therefore, the values reported represent both glycerol and triglycerides as noted in the figure. Our in-house triglyceride measurements involve an initial glycerol extraction step before quantification of triglycerides, and therefore, the values reported represent triglyceride levels.

### Quantification of *E. coli* O21:H+ and *S. aureus* in mouse tissues

For quantification of pathogen in organs, CFUs were quantified. The liver, spleen, kidney, heart, and lung were harvested and homogenized in sterile 1x PBS with 1% Triton X-100 using a BeadMill 24 bench-top bead-based homogenizer (Thermo Fisher Scientific). The spleen, kidney, heart, and lung were homogenized in 1 ml, while liver was homogenized in 500 μl. Homogenates were serially diluted and plated on LB agar and EMB-AVNM agar and incubated at 37°C. Colonies were quantified the following day. The limit of detection for all organs was 100 CFUs.

### Bacterial growth curves

*E. coli* and *S. aureus* cultures were grown overnight in the same conditions described for infection inoculum preparation. These cultures were then diluted 1:100 in 200-μl LB in a 96-well plate with varying concentrations of fluoxetine. The plate was grown for 12 hours at 37°C with shaking, and OD readings were taken at 600 nm every 15 min.

### Serotonin quantification

Serum from mice was harvested by cardiac puncture along with the liver, spleen, lung, and heart. Plasma was collected by tail bleed into heparin-coated microvettes by capillary action. Organs were homogenized in sterile 1x PBS with 1% Triton X-100 using a BeadMill 24 bench-top bead-based homogenizer (Thermo Fisher Scientific). Serotonin levels in serum and organ homogenate were measured using a serotonin ELISA kit (Abcam). Plates were read on a VersaMax microplate reader manufactured by Molecular Devices, and data analysis was done using SofMax Pro. Organ serotonin levels were normalized to total organ weight.

### Platelet flow

Blood (2 μl) was collected by tail bleed throughout infection into microvettes preloaded with 2 μl of anticoagulant citrate dextrose solution (ACD-A) anticoagulant (0.73% citric acid, 2.2% sodium citrate, and 2.45% dextrose). Samples were then immediately transferred into 200 μl of 1x PBS + 2% fetal bovine serum (FBS) on ice. Once all samples were collected, cells were washed once with 1x PBS and then incubated in 25 μl of Brilliant Stain Buffer (BD Biosciences) + extracellular stains for 20 min on ice. Cells were then washed in 1x PBS and fixed in 1x PBS + 1% paraformaldehyde for 10 min on ice. Cells were then washed and resuspended in 1x PBS with 5 μl of counting beads (Invitrogen) for raw count normalization. Cells were analyzed on a Canto II (BD) using FACSDiva software, and data were analyzed using FlowJo software (Tree Star).

### Cytokine ELISAs

Serum from mice was harvested by cardiac puncture. ELISAs were performed to quantify the levels of IL-1β, IL-6, TNFα, and IL-10. ELISA antibodies used are listed in the Key Resources Table. Briefly, NUNC 96-well plates were incubated with primary antibody (50 μl per well; final concentration: IL-1β, 3 μg/ml; IL-6, 3 μg/ml; TNFα, 2 μg/ml; IL-10, 5 μg/ml) in 0.1 M sodium phosphate pH 8.0 at 4°C overnight, washed three times with 1x PBS + 0.1% Tween 20, and then blocked for 1 hour at 37°C with 1x PBS (100 μl per well) + 1% Triton X-100 + 1% BSA. After washing once, 50 μl of samples and standards (diluted in 1x PBS + 1% TritonX-100) were loaded and incubated at 37°C for 1 hour. Following four washes, secondary antibody (50 μl per well; final concentration: IL-1β, 500 ng/ml; IL-6, 500 ng/ml; TNFα, 1 μg/ml; IL-10, 500 ng/ml) was added in 1x PBS + 1% Triton X-100 + 1% BSA, and the plate was incubated for 30 min at 37°C. After washing three times, 1:1000 streptavidin horseradish peroxidase (HRP) (50 μl per well) in 1x PBS + 1% Triton X-100 + 1% BSA was added, and the plate was incubated for 15 min at 37°C. Following three more washes, an o-phenylenediamine dihydrochloride (OPD)-based developing solution [50 μl per well; 5 ml of citrate buffer (0.1 M sodium phosphate and 0.02 M citric acid), 1 tablet of OPD, and 2 μl of 30% H_2_O_2_] was applied to the plate, and the reaction was stopped after 15 min at room temperature using 20 μl of 3 M HCl. The OD_490nm_ was determined using a SpectraMax (Molecular Devices) plate reader and analyzed using SoftMax Pro software 5.4.

### Quantitative reverse transcription polymerase chain reaction

Livers and hearts were harvested and snap-frozen in liquid nitrogen and then stored at −80°C. Frozen tissues were ground into a powder using a mortar and pestle-chilled in liquid nitrogen. RNA was isolated from ~20 mg of tissue powder using QIAGEN Allprep DNA/RNA Mini Kit. Briefly, the sample was lysed in 600 μl of RLT buffer containing 10 μl of β-mercaptoethanol using a BeadMill 24 bench-top bead-based homogenizer (Thermo Fisher Scientific) and centrifuged for 3 min at 18,000*g* in a microcentrifuge. The supernatant was transferred to an AllPrep DNA column and centrifuged for 30 s at 18,000*g*. Flow-through was mixed with 70% ethanol and transferred to an RNeasy spin column and centrifuged for 30 s. The flow-through was discarded, and the column containing RNA was washed with 350 μl of RW1 buffer. Flow-through was discarded. The column was treated with QIAGEN ribonuclease (RNase)–free deoxyribonuclease 1 (DNase 1) [10 μl of DNase 1 (2.73 Kunitz units/μl] and 70 μl of buffer RDD] for 15 min at room temperature. The column was washed again with 350 μl of RW1, and the flow-through was discarded. The column was then washed with 500 μl of RPE buffer. The flow-through was discarded, and the column was spun again to remove any residual wash buffer. After the final spin, the column was placed in a new Eppendorf tube, and RNA was eluted with 40 μl of RNase-free water. cDNA was made using the SuperScript IV kit (Invitrogen) after all samples were diluted to 20 ng/μl. Real-time quantitative polymerase chain reaction (qPCR) was performed using iTaq SYBR Green Mix (Bio-Rad) on a Thermo Fisher QuantStudio 5 qPCR machine. Relative standard curves were generated by mixing cDNA from each sample and then making serial dilutions of the mix. The analysis of gene expression was then done by comparing threshold cycle (Ct) of the gene of interest to the relative standard curve for that gene and then normalizing this to the expression of the housekeeping gene (*Rps17*) for each sample. See Table 1 for primer sequences.

### Peritoneal lavage flow cytometry preparation

Peritoneal lavage was collected immediately after euthanasia. Briefly, the mouse skin was peeled back to expose the peritoneal cavity. The 1x PBS (5 ml) was injected into the peritoneal cavity, taking care not to puncture any internal organs. The mouse was then shaken for 10 s, and then the lavage fluid was taken back up into the syringe. The lavage fluid was then mixed with 10 ml of RPMI complete media [RPMI GlutaMAX, 1% penicillin-streptomycin, 10% heat-inactivated FBS, 1% Hepes (1 M), 1% sodium pyruvate, and 1% MEM-non essential amino acid solution (MEM-NEAA)] on ice. Once all samples were collected, they were centrifuged at 400*g* for 4 min and the supernatant was poured off. The Ack lysis buffer (2 ml) was added, and samples were incubated for 2 min before 10 ml of RPMI complete media was added. Samples were again centrifuged at 400*g* for 4 min. Supernatant was discarded, and samples were transferred to a 96-well v-bottom plate.

### Liver flow cytometry preparation

Livers were harvested and transferred into 10 ml of RPMI complete media on ice. Livers were mashed through a 100 μM mesh filter using the plunger from a 5-ml syringe. Cells were centrifuged at 60*g* for 2 min at 4°C with no brake. The supernatants were then transferred to fresh conical tubes and centrifuged at 400*g* for 4 min at 4°C. Supernatants were then removed by vacuum, and pellets were resuspended in 43% Percoll in 1x PBS. Samples were then centrifuged at 850*g* for 25 min at 4°C with no brake. Supernatants were then removed by vacuum, pellets were resuspended in 2 ml of Ack lysis buffer, and samples were incubated for 2 min before 10-ml RPMI complete media was added. Samples were again centrifuged at 400*g* for 4 min. Supernatant was discarded, and samples were transferred to a 96-well v-bottom plate.

### Spleen flow cytometry preparation

Spleens were harvested and transferred into 10 ml of RPMI complete media on ice. Spleens were mashed through a 40 μM mesh filter using the plunger from a 5-ml syringe into RPMI with DNase 1 (1 U/ml; Roche). Cells were centrifuged at 400*g* for 4 min. Supernatants were then removed by vacuum, pellets were resuspended in 2 ml of Ack lysis buffer, and samples were incubated for 2 min before 10-ml RPMI complete media was added. Samples were again centrifuged at 400*g* for 4 min. Supernatant was discarded, and samples were transferred to a 96-well v-bottom plate.

### Flow cytometry cell incubation, staining, and analysis

Samples in a 96-well v-bottom plate were resuspended in 200-μl RPMI complete media +1:1000 Brefeldin A. The plate was incubated at 37°C and 5% CO_2_ for 4 hours. Following incubation, cells were washed once in 1x PBS and then treated with 25 μl of 1x PBS + 1:100 Fc block +1:100 Zombie ultraviolet for 10 min on ice. Cells were then washed in 1x PBS and then incubated in 25 μl of Brilliant Stain Buffer (BD Biosciences) + extracellular stains for 20 min on ice. Cells were then washed in 1x PBS and then fixed in 200 μl of 1% formaldehyde overnight at 4°C. The following day, cells were washed with 1x PBS and stained for 30 min with intracellular stains in 50 μl of saponin permeabilization buffer (Invitrogen). The cells were then washed again and resuspended in 200 μl of PBS. A 5 μl of counting beads (Invitrogen) was added to each sample for raw count normalization. Cells were analyzed on a LSR II (BD) using FACSDiva software, and data were analyzed using FlowJo software (Tree Star).

### Organ triglyceride measurement

Hearts were removed from the body cavity, blood was drained from the heart chambers, and then the organs were snap-frozen in liquid nitrogen. Livers were removed from body, weighed, and snap-frozen in liquid nitrogen. Frozen organs were ground into a powder using a mortar and pestle-chilled in liquid nitrogen. Lipids were extracted from ~20 mg of organ powder using a lipid extraction kit (Abcam). Briefly, powder was homogenized in 375 μl of extraction buffer using a BeadMill 24 bench-top bead-based homogenizer (Thermo Fisher Scientific). The homogenate was then transferred to a fresh 1.5-ml Eppendorf tube and centrifuged at 10,000*g* for 5 min at 4°C. A total of 250 μl was then transferred into a PCR strip. The samples were then allowed to evaporate overnight at 37°C. The following day, samples were resuspended in 50 μl of suspension buffer by vigorous sonication and vortexing. Triglycerides were measured using Fujifilm L-Type Triglyceride M kit, following the manufacturer’s protocol.

### Western blot

Heart tissue powder was homogenized in 600 μl of radioimmunoprecipitation assay (RIPA) buffer supplemented with 1:100 protease inhibitor cocktail (Sigma-Aldrich) and 1:100 halt protease and phosphatase inhibitor cocktail (Thermo Fisher Scientific). Lysates were centrifuged at 4°C for 30 min at 18,000*g* and transferred to a new tube. Protein concentration was quantified with a bicinchoninic acid assay (BCA) reaction. Samples were diluted in RIPA + a 1:10 mixture of 2-mercaptoethanol and NuPAGE LDS Sample Buffer (4X) (Invitrogen) to a final protein concentration of 1 μg/μl, then incubated at 70°C for 10 min, and sonicated in a water bath for 10 min. A total of 15 μl of each sample was loaded into 7% NuPage 1.0 mm × 12-well tris-acetate gels with tris-acetate SDS running buffer (50 ml of 20X tris-acetate SDS in 950 ml of deionized H_2_O for 60 min at 150 V). Gels were placed on Trans-Blot Turbo™ Midi 0.2 μm Nitrocellulose Transfer Packs (Bio-Rad) and transferred in Trans-Blot Turbo Transfer System (Bio-Rad) (2.5 A, 25 V for 10 min). Membranes were stained with Ponceau Total Protein Stain (Prometheus) to visualize the total protein, and then membranes were cut into strips for each protein to be assayed. The strips were then washed with 1X Tris-buffered saline with Tween 20 (TBST) to destain and then placed into blocking solution (5% BSA in 1X TBST) on a shaker platform for 1 hour at room temperature. Blocking solution was removed, and primary antibody solutions were added to membranes (1:1000 antibody in Prometheus OneBlock Western-CL Blocking Buffer) and placed on shaker platform overnight at 4°C. Primary antibody solutions were removed, and membranes were washed on shaker platform for 5 min with 1X TBST, repeated four times at room temperature (RT). Secondary antibody solution was added containing 1:3000 anti-rabbit immunoglobulin G (IgG) HRP-linked antibody in blocking buffer (5% BSA in 1X TBST), and membranes were placed on shaker platform for 1 hour at RT. Secondary antibody solutions were removed, and membranes were washed again on a shaker platform for 5 min with 1X TBST, repeated four times at RT. Nitrocellulose blots were developed using a mixture of Femto and Dura chemiluminescent reaction and visualized with a Bio-Rad Gel Doc XR+ machine. For blots targeting total and phosphorylated forms of the same protein, the same lysate was run on two different gels at the same time in the same gel rig. The gels were then transferred to the same membrane. After staining of the membrane with Ponceau, the membranes were cut and destained. The cut membranes were then probed for the relevant protein. Total and phosphorylated blots had their own cyclophilin A loading control that was run on the same gel. The Bio-Rad image lab software was used to quantify bands.

### Lipid tolerance test

Mice were retro-orbitally injected with 100 μl of intralipid (Sigma-Aldrich, 20% emulsion) at a dose of 10 ml/g body weight 7 hours post-infection. Blood was collected via the lateral tail vein at 0, 15, 30, 45, and 60 minutes post-injection. Blood was centrifuged at 6000 rpm for 20 min, and serum was collected for triglyceride measurements. Total triglycerides were measured using Fujifilm L-Type Triglyceride M kit, following the manufacturer’s protocol.

### Pluronic test

To make pluronic solution, 5 g of pluronic (Sigma-Aldrich, Pluronic F-127) was dissolved in 50 ml of water, and the solution was filter sterilized with a 0.22-μm filter. Mice were intraperitoneally injected with the pluronic solution at a dose of 10 ml/g bodyweight at the time of infection. Blood was collected by tail vein at 0, 60, 120, 180, and 240 min post-injection. Blood was spun at 6000 rpm for 20 min, and serum was collected for triglyceride measurements. Triglycerides were measured using Fujifilm L-Type Triglyceride M kit, following the manufacturer’s protocol.

### Lomitapide treatment

Mice were orally gavaged with lomitapide (10 mg/kg) in 2% dimethyl sulfoxide (DMSO) + corn oil or administered 2% DMSO + corn oil vehicle, daily for 3 days pre-infection.

### U-^13^C glucose tracing

We followed the methods described previously (*79*, *80*). Mice were pretreated with vehicle or fluoxetine as noted above and infected with polymicrobial sepsis as described above. For IgG/anti-IL10R and pluronic conditions, mice were treated as described above. Food was removed at the time of infection for both infected and uninfected conditions. At 7 hours post-infection, mice were gavaged with U-^13^C glucose (50 mg), and hearts were collected at 15 min post-gavage, snap-frozen in liquid nitrogen, and stored at −80°C. For tissue metabolite extraction, hearts were ground to a powder in liquid nitrogen with a mortar and pestle, and ~20 mg of ground tissue was homogenized with 500 μl of methanol, 500 μl of high-performance liquid chromatography grade H_2_O water and spiked with 2 nmol of norvaline. A 100-μl aliquot of homogenate was taken to determine tissue protein concentration using BCA protein assay (Thermo Fisher Scientific). The remaining homogenate was transferred to a 2-ml Eppendorf tube, and 1 ml of chloroform was added. Samples were vortexed for 5 min and centrifuged for 5 min at 4°C at 21,000*g*. The polar phase was collected and dried under vacuum.

Derivatization was performed using a Gerstel MPS with 15 μl of 2% (w/v) methoxyamine hydrochloride (Thermo Fisher Scientific) in pyridine and with *N*-tertbutyldimethylsilyl-*N*-methyltrifluoroacetamide with 1% *tert*butyldimethylchlorosilane (Regis Technologies). Polar derivatives were analyzed by gas chromatography–mass spectrometry using a DB-35MS column (30 m–by–0.25 mm internal diameter × 0.25 μm, Agilent J&W Scientific) installed in an Agilent 7890B gas chromatograph interfaced with an Agilent 5977B MSD mass spectrometer. The isotopolog distribution was determined by integration, and natural isotope abundance was corrected using an in-house MATLAB-based script.

### Statistical analysis

Statistical tests were done using Prism version 8.4.3. Sample sizes, number of independent experiments pooled, statistical tests used, and *P* values for each figure and panel are indicated in the figure legends.
