# Supplementary material for: Fluoxetine promotes IL-10–dependent metabolic defenses to protect from sepsis-induced lethality
Source: Sci Adv. 2025 Feb 14;11(7):eadu4034. doi: 10.1126/sciadv.adu4034 (PMC11827869; doi:10.1126/sciadv.adu4034)
Supplement: Supplementary file 1 — Figs. S1 to S7 Table S1 [file sciadv.adu4034_sm.pdf]

Science Advances
AAAS

Supplementary Materials for

Fluoxetine promotes IL-10–dependent metabolic defenses to protect from sepsis-induced lethality

Robert M. Gallant *et al.*

Corresponding author: Janelle S. Ayres, jayres@salk.edu

Sci. Adv. **11**, eadu4034 (2025)
DOI: 10.1126/sciadv.adu4034

This PDF file includes:

Figs. S1 to S7
Table S1

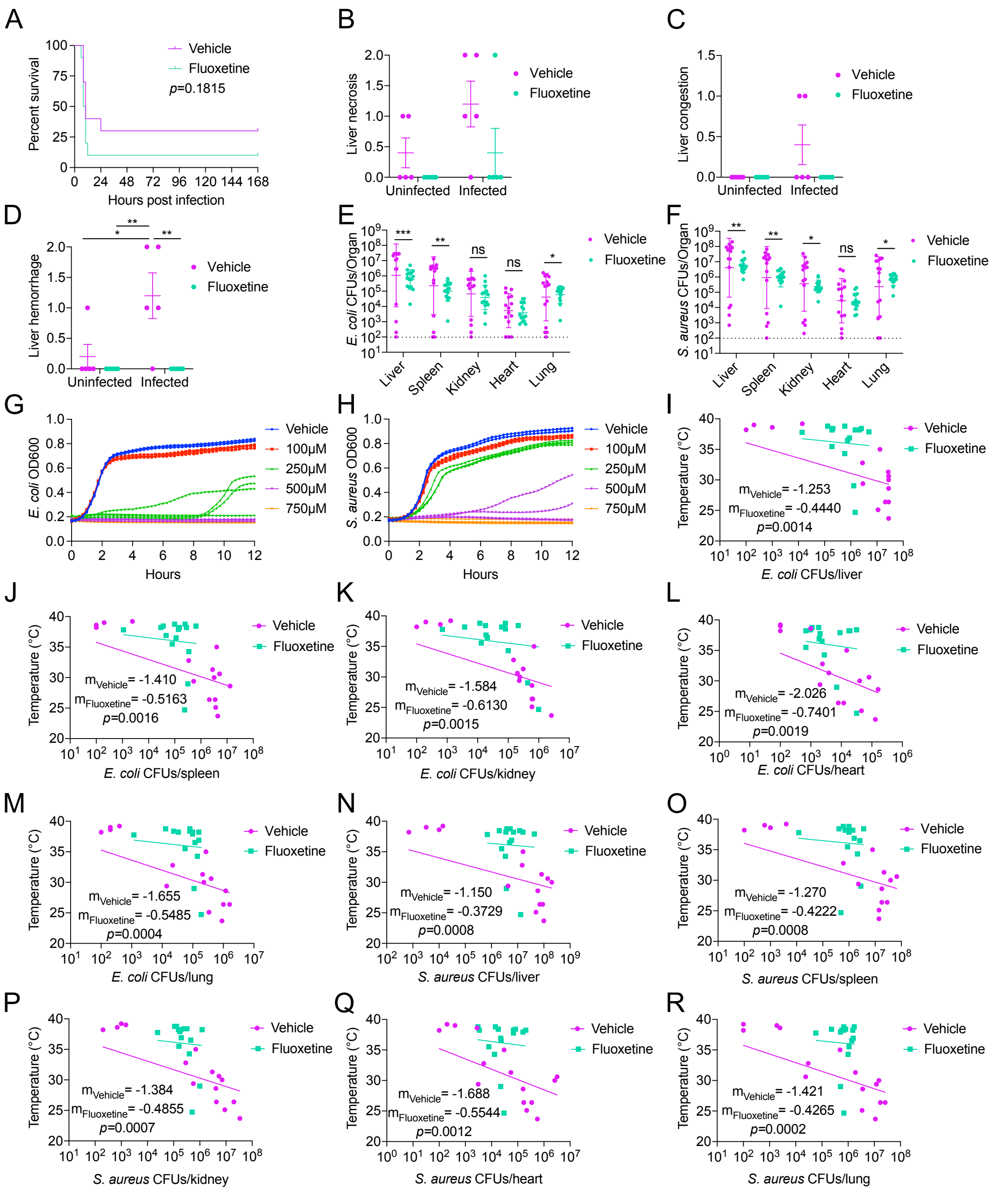

Supplemental Figure 1: Fluoxetine pre-treatments protects from polymicrobial sepsis. (A) Survival of mice treated with vehicle or 40mg/kg fluoxetine 30 minutes post-infection. n = 10 mice per condition. Two replicates combined. Log rank analysis. (B-D) Scores from histolopathology analysis of H&E stained livers at 10 hours post infection in vehicle or fluoxetine pre-treated mice infected with polymicrobial sepsis. n = 5 per condition, one independent experiment shown. Data represent mean ± SEM. Two-way ANOVAs with Tukey's multiple comparisons. (E-F) *E. coli* or *S. aureus* burden analysis from vehicle or fluoxetine pre-treated mice at 8-10 hours post infection. n = 15 per condition, three experiments combined. Data represent geometric mean ± geometric SD. Unpaired t-tests. (G-H) *E. coli* or *S. aureus* growth curves grown in varying concentrations of fluoxetine. n = 4 per condition, one representative experiment out of two independent replicates shown. (I-R) Reaction norm analyses plotting body temperature at the time of dissection against *E. coli* or *S. aureus* CFUs from mice in Figure 2. (I) *E. coli* liver, (J) *E. coli* spleen, (K) *E. coli* kidney, (L) *E. coli* heart, (M) *E. coli* lung, (N) *S. aureus* liver, (O) *S. aureus* spleen, (P) *S. aureus* kidney, (Q) *S. aureus* heart, (R) *S. aureus* lung. n = 15 per condition, three independent experiments combined. Semilog linear regression, y-intercept constrained to uninfected temperature for each pre-treatment condition, Extra sum-of-squares F Test to compare slopes. * $p<0.05$, ** $p<0.01$, *** $p<0.001$.

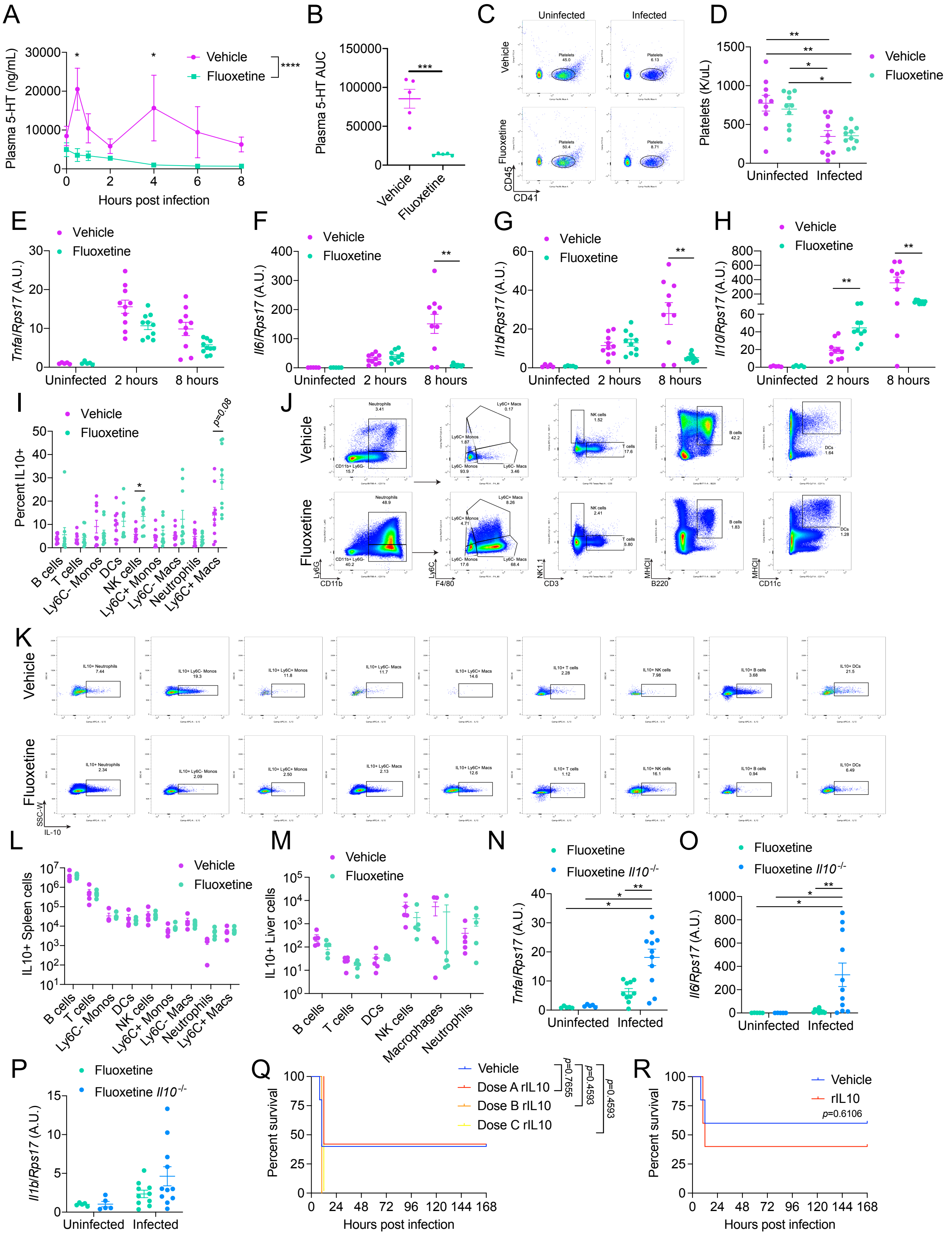

Supplemental Figure 2: Fluoxetine regulates the degree and duration of the inflammatory response during sepsis. (A-B) Time course of plasma serotonin levels. (A) Plasma serotonin levels throughout infection of mice pre-treated with vehicle or fluoxetine and infected with polymicrobial sepsis. (B) Area under the curve analysis of (A). n = 5 per condition, one representative experiment shown out of two. For time-course, two-way ANOVA with Dunnett's multiple comparisons. For AUC, unpaired t-test. (C-D) Flow cytometry analysis of platelets. (C) Gating strategy and (D) Platelet count at 8 hours post infection of mice pre-treated with vehicle or

fluoxetine and infected with polymicrobial sepsis. n = 10 per condition, two independent experiments combined. Two-way ANOVA with Tukey's multiple comparisons. (E-H) Hepatic transcript levels of (E) *Tnfa*, (F) *Il6*, (G) *Il1b*, and (H) *Il10* in vehicle or fluoxetine pre-treated mice infected with polymicrobial sepsis. n=5-15 per condition, three independent experiments combined. Unpaired t-tests with Holm-Sidak multiple comparisons correction. (I) Percentage of indicated population that was IL10+, same mice as panel 4E. Unpaired t-tests with Holm-Sidak multiple comparisons correction. n=10 mice per condition, two replicates combined. (J-K) Gating strategies for panel 4E. (L-M) Flow cytometry analysis of cells isolated from the (L) spleen and (M) liver 2 hours post infection from vehicle or fluoxetine pre-treated mice infected with polymicrobial sepsis. n=5 per condition, one experiment shown. (N-P) Hepatic transcript levels of (K) *Tnfa*, (L) *Il6*, and (M) *Il1b* at 10 hours post infection in wildtype or $Il10^{-/-}$ mice pre-treated with fluoxetine infected with polymicrobial sepsis. n=5-11 per condition, two independent experiments combined. Two-way ANOVA with Tukey's multiple comparisons test. (Q) Survival of mice infected with polymicrobial sepsis and treated intraperitoneally with either Dose A rIL10 (1ug at the time of infection), Dose B rIL10 (1ug at 4hrs post-infection), Dose C (0.5ug every hr post-infection) or vehicle. n=5 mice per condition, one experimental replicate shown. Log-rank analysis. (R) Survival of mice infected with polymicrobial sepsis and treated with 1ug of rIL10 or vehicle intraperitoneally at 30 min, 2 hr and 8 hr post-infection. n=5 mice per condition, one experimental replicate shown. Log-rank analysis. In all panels data represent mean ± SEM. * p<0.05, ** p<0.01, *** p<0.001.

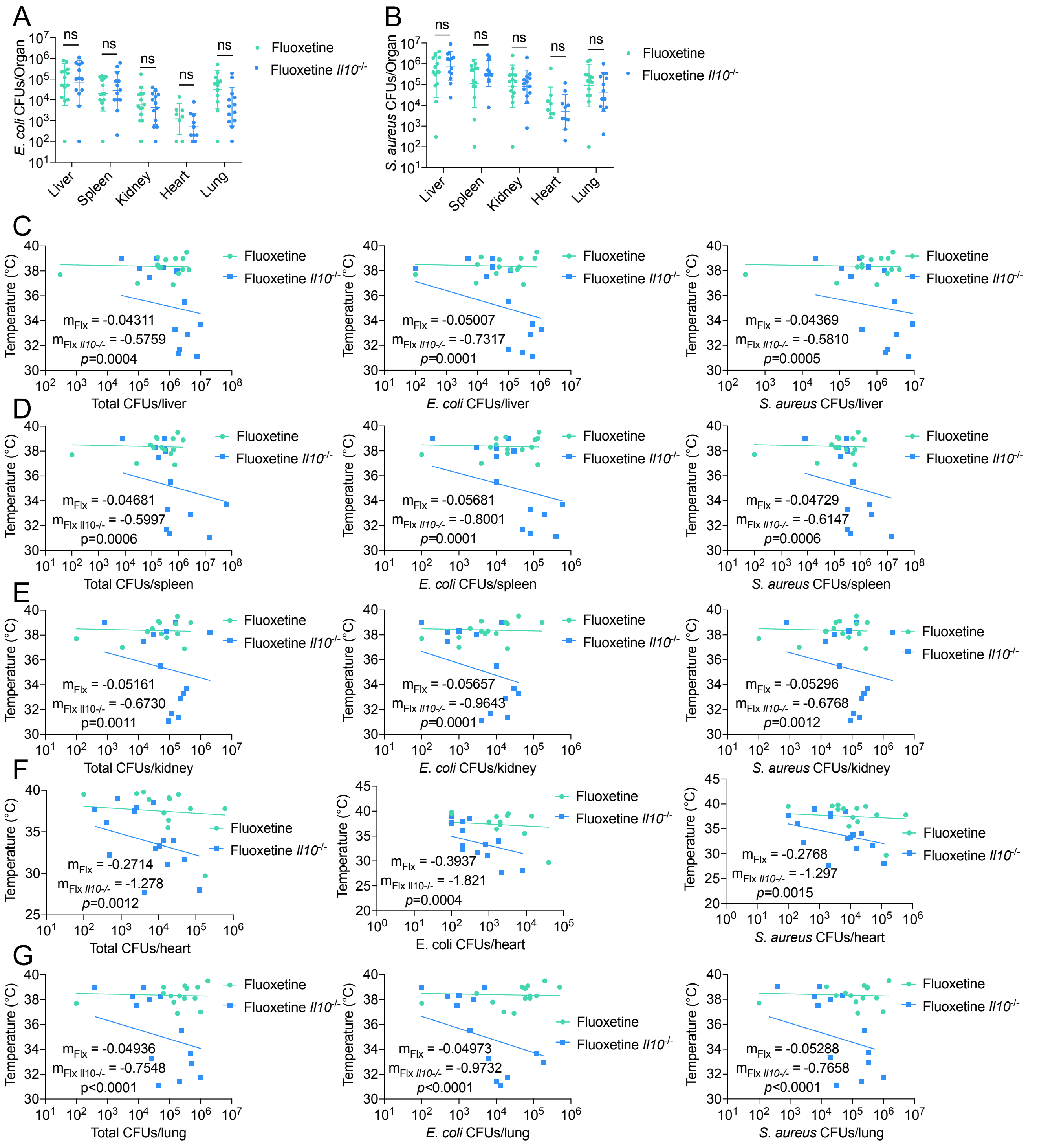

Supplemental figure 3: IL-10 is required for fluoxetine-mediated cooperative defenses during sepsis. (A-B) (A) *E. coli* and (B) *S. aureus* burden analysis at 10 hours post infection from wildtype or *Il10*$^{-/-}$ mice treated with fluoxetine infected with polymicrobial sepsis. Data represent geometric mean ± geometric SD. Same mice as in panel 4M. n=8-14 per condition, two-three independent experiments combined, unpaired t-tests. (C-G) Reaction norm analyses plotting body temperature at the time of dissection against total, *E. coli*, or *S. aureus* CFUs. (C) Liver, (D) Spleen, (E) Kidney, (F) Heart, (G) Lung. Same mice as in panel 4M. n=8-14 per condition, two-three independent experiments combined, semilog linear regression, y-intercept constrained to uninfected temperature of each pre-treatment condition, Extra sum-of-squares F Test to compare slopes.

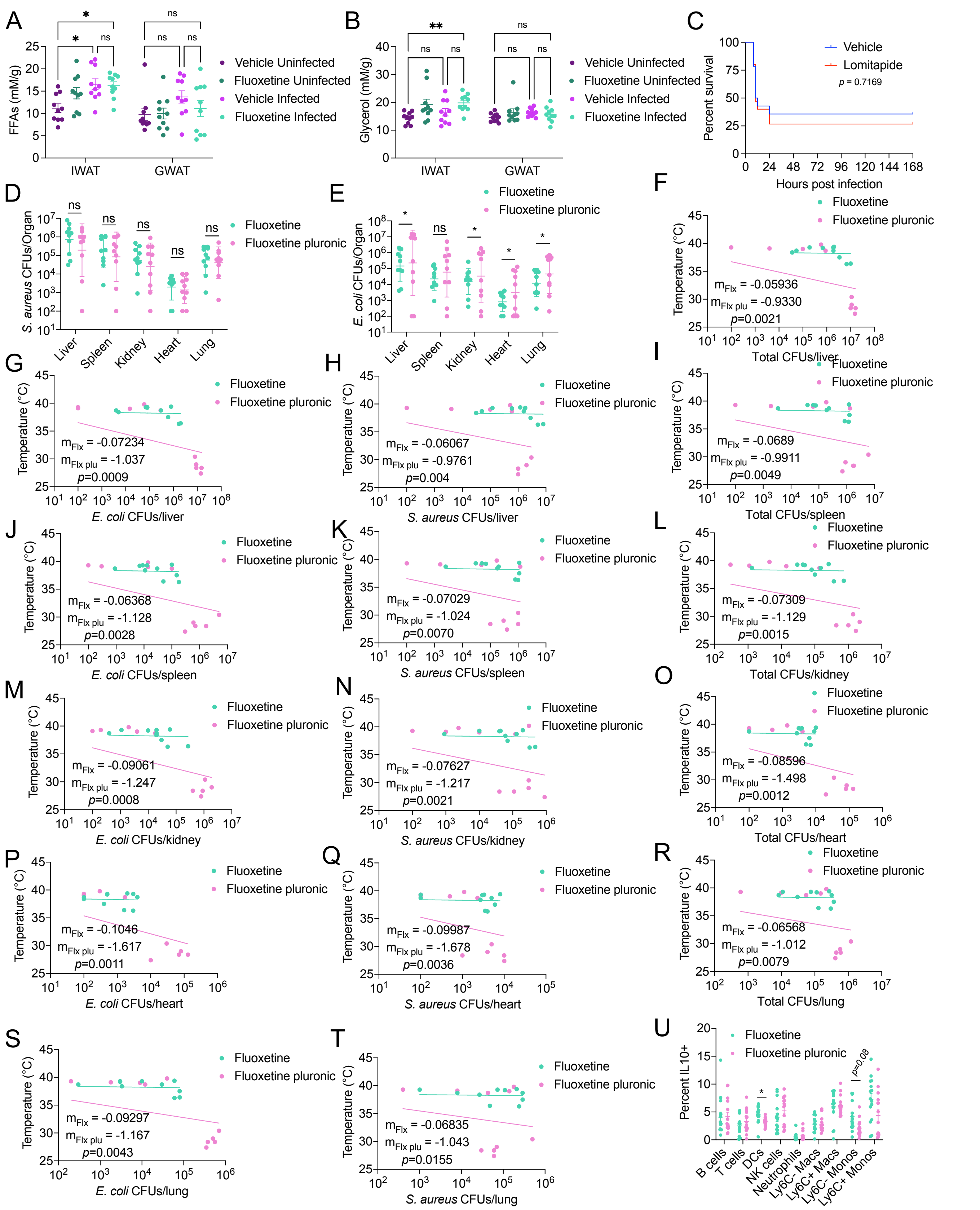

Supplemental figure 4: Lpl activity is required for fluoxetine-mediated defenses during sepsis. (A-B) Ex vivo lipolysis assay of the IWAT and GWAT at 2 hrs post-infection. (A) Free fatty acid levels. (B) glycerol levels. n = 10 mice per condition. Error bars +/- SEM. Two-way ANOVA with Tukey's multiple comparisons test. Two independent experiments combined. (C) Survival of vehicle and lomitapide treated mice infected with polymicrobial sepsis. n=15 per condition, two independent experiments combined. Log-rank analysis.

(D-E) (D) *S. aureus* or (E) *E. coli* burden analysis at 8-10 hours post infection in fluoxetine pre-treated mice with or without Pluronic F-127 injection at the time of infection with polymicrobial sepsis. Data represent geometric mean ± geometric SD. Same mice as in panel 5I. n=10 per condition, two independent experiments combined. Unpaired t-tests. (F-T) Reaction norm analyses plotting body temperature at the time of dissection against total, *E. coli*, or *S. aureus* CFUs. (F-H) Liver, (I-K) Spleen, (L-N) Kidney, (O-Q) Heart, (R-T) Lung. Same mice as in panel 5J. n=10 per condition, two independent experiments combined. Semilog linear regression, y-intercept constrained to uninfected temperature, Extra sum-of-squares F Test to compare slopes. (U) Peritoneal lavage flow cytometry at 2 hours post infection in fluoxetine treated mice injected with Pluronic F-127 at the time of infection with polymicrobial sepsis. n=15 per condition, two independent experiments combined, unpaired t-tests. In all panels data represent mean ± SEM. * $p<0.05$, ** $p<0.01$

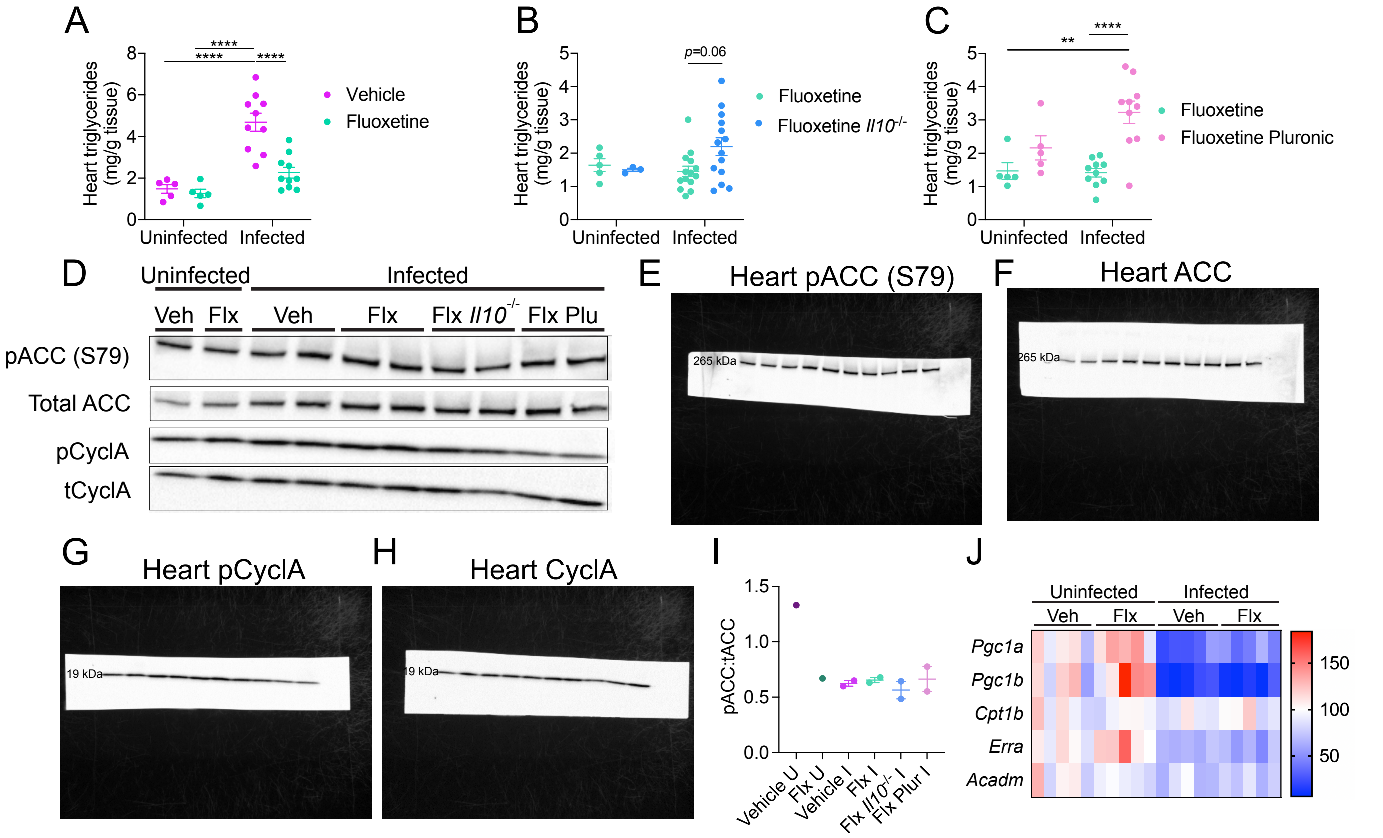

Supplemental Figure 5: Fluoxetine protects from sepsis induced impairment of cardiac glucose oxidation. (A) Cardiac triglyceride levels at 8-10 hours post infection in vehicle or fluoxetine pre-treated mice infected with polymicrobial sepsis. n=5-10 per condition, two independent experiments combined. Two-way ANOVA with Tukey's multiple comparisons. (B) Cardiac triglycerides at 8-10 hours post infection in fluoxetine pre-treated wildtype or *Il10*$^{-/-}$ mice infected with polymicrobial sepsis. n=3-14 per condition, three independent experiments combined. Two-way ANOVA with Tukey's multiple comparisons. (C) Cardiac triglycerides at 8-10 hours post infection in fluoxetine pre-treated mice injected with water or Pluronic F-127 at the time of infection with polymicrobial sepsis. n=5-10 per condition, two independent experiments combined. Two-way ANOVA with Tukey's multiple comparisons. (D) Western blot of acetyl-coa carboxylase (ACC), phospho-ACC, and cyclophilin A of hearts at 8-10 hours post infection in uninfected vehicle or fluoxetine pre-treated WT mice, infected vehicle or fluoxetine pre-treated WT mice, infected-fluoxetine pre-treated *Il10*$^{-/-}$ mice, and infected-fluoxetine pre-treated Pluronic F-127 injected mice at the time of infection. Blot labeled "tCyclA" is the loading control for the total ACC blot. Blot labeled "pCyclA" is the loading control for the pACC blot. n=1-2 per condition, representative of three independent experiments. (E-H) Uncropped blots for (D)**.** (I) Quantification for (D)**.** (J) Cardiac fatty acid oxidation-related transcript levels at 10 hours post infection in vehicle or fluoxetine pre-treated mice infected with polymicrobial sepsis. n=5 per condition, one representative experiment shown out of two replicates. Two-way ANOVA with Tukey's multiple comparisons, no significant results comparing infected vehicle to infected fluoxetine. In all panels data represent mean ± SEM. * p<0.05, ** p<0.01, *** p<0.001, **** p< 0.0001.

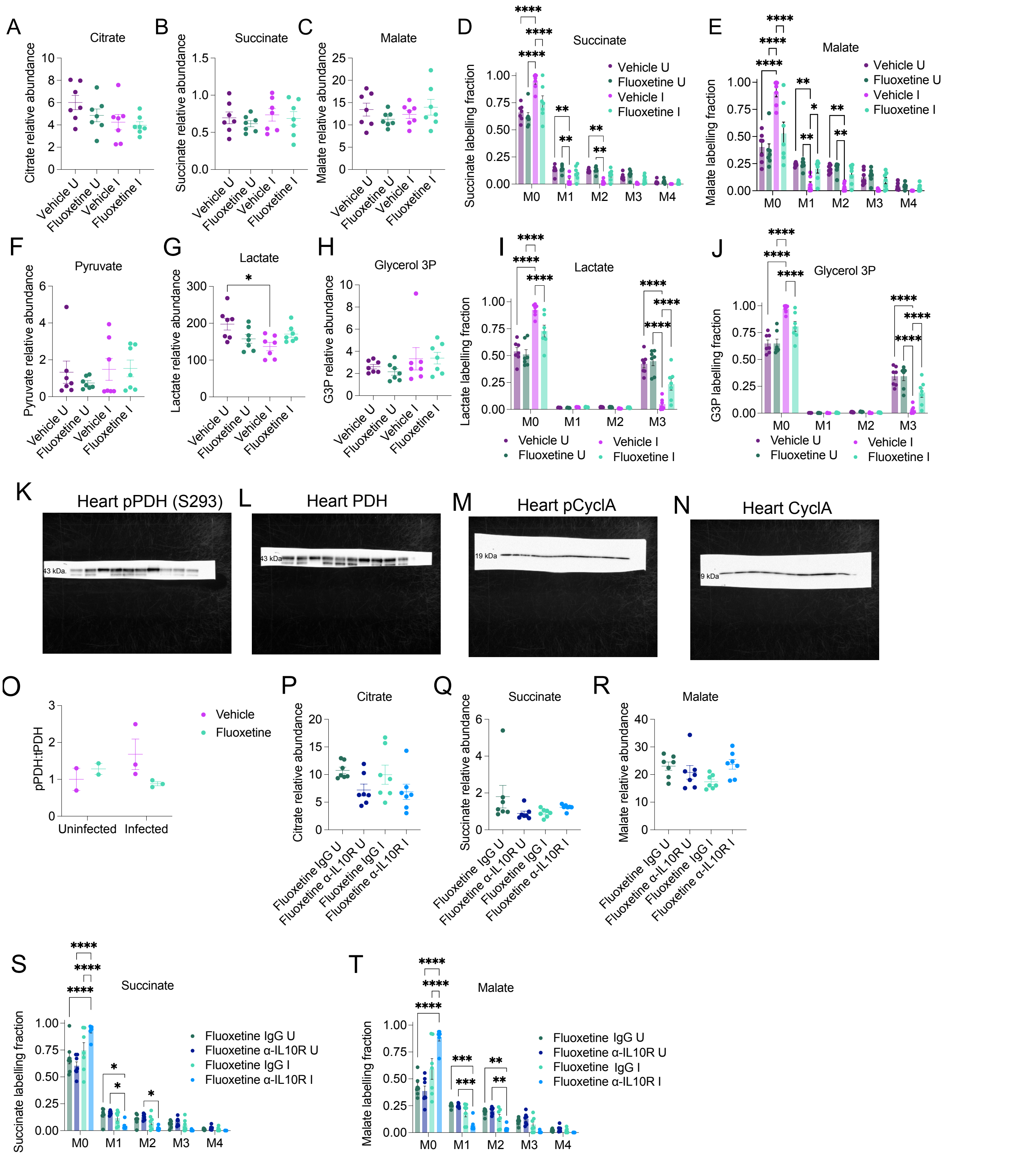

Supplemental Figure 6: Fluoxetine protects from sepsis induced impairment of cardiac glucose oxidation in an IL10 dependent manner. (A-C) Relative abundance of (A) citrate (B) succinate and (C) malate in the hearts at 7 hrs post-infection of vehicle and fluoxetine pre-treated infected and uninfected mice gavaged with U-^{13}C glucose. n = 7 mice per condition. One experiment shown. (D-E) Labeling fraction of (D) succinate and (E) malate in the hearts at 7 hrs post-infection of vehicle and fluoxetine pre-treated infected and uninfected mice gavaged with U-^{13}C glucose. n = 7 mice per condition. One experiment shown. Two-way ANOVA with Tukey's multiple comparisons. (F-H) Relative abundance of (F) pyruvate (G) lactate and (H) glycerol 3P in the hearts at 7 hrs post-infection of vehicle and fluoxetine pre-treated infected and uninfected

mice gavaged with U-^{13}C glucose. n = 7 mice per condition. One experiment shown. (I-J) Labeling fraction of (I) lactate and (J) glycerol 3P in the hearts at 7 hrs post-infection of vehicle and fluoxetine pre-treated infected and uninfected mice gavaged with U-^{13}C glucose. n = 7 mice per condition. One experiment shown. Two-way ANOVA with Tukey's multiple comparisons. (K-O) Original blots and quantification for Figure 7E.
(P-R) Relative abundance of (P) citrate (Q) succinate and (R) malate in the hearts at 7 hrs post-infection of fluoxetine pre-treated infected and uninfected mice gavaged with U-^{13}C glucose and injected with anti-IL10R or isotype control. n = 7 mice per condition. One experiment shown. (S-T) Labeling fraction of (S) succinate and (T) malate in the hearts at 7 hrs post-infection of fluoxetine pre-treated infected and uninfected mice gavaged with U-^{13}C glucose and injected with anti-IL10R or isotype control. n = 7 mice per condition. One experiment shown. Two-way ANOVA with Tukey's multiple comparisons. In all panels data represent mean ± SEM. * $p<0.05$, ** $p<0.01$, *** $p<0.001$, **** $p< 0.0001$.

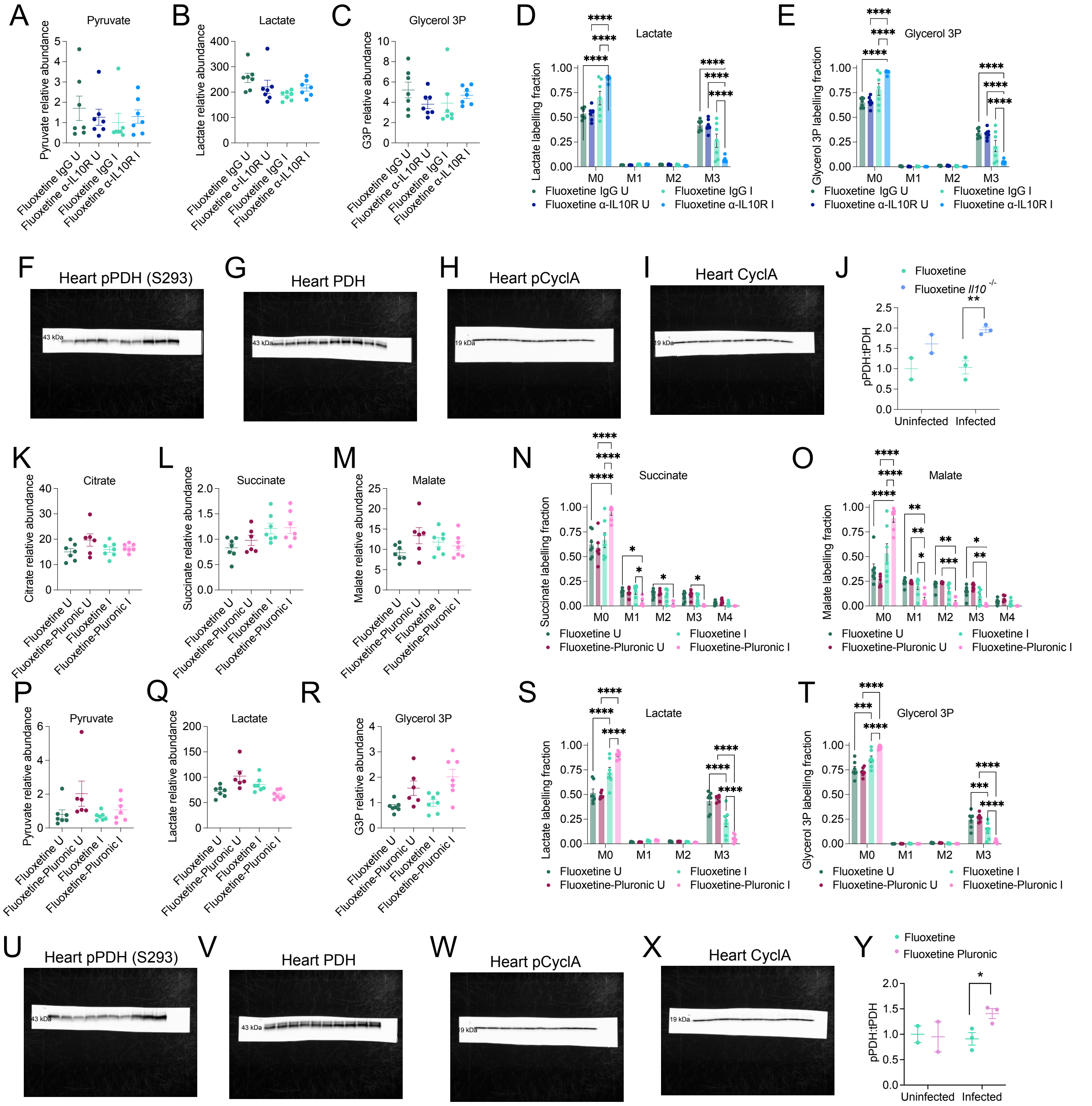

Supplemental Figure 7: Fluoxetine protects from sepsis induced impairment of cardiac glucose oxidation in an LPL dependent manner. (A-C) Relative abundance of (A) pyruvate (B) lactate (C) glycerol 3P in the hearts at 7 hrs post-infection of fluoxetine pre-treated infected and uninfected mice gavaged with U-^{13}C glucose and injected with anti-IL10R or isotype control. n = 7 mice per condition. One experiment shown. (D-E) Labeling fraction of (D) lactate and (E) glycerol 3P in the hearts at 7 hrs post-infection of fluoxetine pre-treated infected and uninfected mice gavaged with U-^{13}C glucose and injected with anti-IL10R or isotype control. n = 7 mice per condition. One experiment shown. Two-way ANOVA with Tukey's multiple comparisons. (F-J) Original blots and quantification for Figure 7I. (K-M) Relative abundance of (K) citrate (L) succinate and (M) malate in the hearts at 7 hrs post-infection of fluoxetine pre-treated infected and uninfected mice gavaged with U-^{13}C glucose and injected with water or pluronic at the time of infection. n = 6 mice per condition. One experiment shown. (N-O) Labeling fraction of (N) succinate and (O) malate in the hearts at 7 hrs post-infection of fluoxetine pre-treated infected and uninfected mice gavaged with U-^{13}C glucose and injected with water or pluronic at the time of infection. n = 6 mice per condition. One experiment shown. Two-way ANOVA with

Tukey's multiple comparisons. (P-R) Relative abundance of (P) pyruvate (Q) lactate and (R) glycerol 3P in the hearts at 7 hrs post-infection of fluoxetine pre-treated infected and uninfected mice gavaged with U-^{13}C glucose and injected with water or pluronic at the time of infection. n = 6 mice per condition. One experiment shown. (S-T) Labeling fraction of (S) lactate and (T) glycerol 3P in the hearts at 7 hrs post-infection of fluoxetine pre-treated infected and uninfected mice gavaged with U-^{13}C glucose and injected with water or pluronic at the time of infection. n = 6 mice per condition. One experiment shown. Two-way ANOVA with Tukey's multiple comparisons. (U-Y) Original blots and quantification for Figure 7L. In all panels data represent mean ± SEM. * p<0.05, ** p<0.01, *** p<0.001, **** p< 0.0001.

	Forward	Reverse
Rps17	CGCCATTATCCCCAGCAAG	TGTCGGGATCCACCTCAATG
Il1b	AAGGGCTGCTTCCAAACCTTTGAC	ATACTGCCTGCCTGAAGCTCTTGT
Il6	CTCTGGAGCCCACCAAGAAC	TTGTGAAGTAGGGAAGGCCG
Tnfa	AGGGGATTATGGCTCAGGGT	GAGTCCTTGATGGTGGTGCA
Il10	GCTGGACAACATACTGCTAACC	ATTTCCGATAAGGCTTGGCAA
Fasn	CAAGTGTCCACCAACAAGCG	GGAGCGCAGGATAGACTCAC
Acaca	GTGGATGGCTTGCGGGAATG	CATCTCCATGTGCCGAGGGT
Srebp1	ACAGGAGGACATCTTGCTGC	AGATCTCTGCCAGTGTTGCC
CD36	TGGCCTTACTTGGGATTGG	CCAGTGTATATGTAGGCTCATCCA
Apob	TACTTCCACCCACAGTCCCCT	CCTTAGAAGCCTTGGGCACAT
Mttp	TCTCACAGTACCCGTTCTT	TCTTCTCCGAGAGACATATCC
Cav1	GCCGCGTCTACTCCATCTAC	CTGATGCGGATGTTGCTGAATA
Lpl	TGGCGTAGCAGGAAGTCTGA	TGCCTCCATTGGGATAAATGTC
Pgc1a	AACCACACCCACAGGATCAGA	TCTTCGCTTTATTGCTCCATGA
Pgc1b	AGGGAGGAAGATGGACAGCT	GTATCCAGGCACATCGAGGG
Cpt1b	CATGTATCGCCGCAAACTGG	CCTGGGATGCGTGTAGTGTT
Erra	GCCAGTCCTGACAGTCCAAA	CATCCTCCTCCTCCTTGTGC
Acadm	TCGGTGAAGGAGCAGGTTTC	TTCGTGGCTTCGTCTAGAGC

Table S1 – List of primers used in this study
